# Six-month effective treatment of corneal graft rejection

**DOI:** 10.1126/sciadv.adf4608

**Published:** 2023-03-22

**Authors:** Tuo Meng, Jinhua Zheng, Min Chen, Yang Zhao, Hadi Sudarjat, Aji Alex M.R., Vineet Kulkarni, Yumin Oh, Shiyu Xia, Zheng Ding, Hyounkoo Han, Nicole Anders, Michelle A. Rudek, Woon Chow, Walter Stark, Laura M. Ensign, Justin Hanes, Qingguo Xu

**Affiliations:** ^1^ Department of Pharmaceutics, Virginia Commonwealth University, Richmond, VA 23298, USA.; ^2^ Department of Ophthalmology, Affiliated Hospital of Guizhou Medical University, Guiyang, Guizhou 550004, China.; ^3^ Eye Institute of Shandong First Medical University, Qingdao Eye Hospital of Shandong First Medical University, Qingdao, Shandong 266073, China.; ^4^ Department of Ophthalmology, The Wilmer Eye Institute, The Johns Hopkins University School of Medicine, 400 North Broadway, Baltimore, MD 21231, USA.; ^5^ Center for Nanomedicine, The Johns Hopkins University School of Medicine, 400 North Broadway, Baltimore, MD 21231, USA.; ^6^ Department of Ophthalmology, Second Xiangya Hospital of Central South University, Changsha, Hunan, China.; ^7^ Department of Medicine, The Johns Hopkins University, Baltimore, MD 21231, USA.; ^8^ Department of Ophthalmology, Virginia Commonwealth University, Richmond, VA 23298, USA.; ^9^ Center for Pharmaceutical Engineering and Institute for Structural Biology, Drug Discovery and Development (ISB3D), Massey Cancer Center, Virginia Commonwealth University, Richmond, VA 23298, USA.

## Abstract

Topical corticosteroid eye drop is the mainstay for preventing and treating corneal graft rejection. While the frequent topical corticosteroid use is associated with risk of intraocular pressure (IOP) elevation and poor patient compliance that leads to graft failure and the requirement for a repeated, high-risk corneal transplantation. Here, we developed dexamethasone sodium phosphate (DSP)–loaded dicarboxyl-terminated poly(lactic acid) nanoparticle (PLA DSP-NP) formulations with relatively high drug loading (8 to 10 weight %) and 6 months of sustained intraocular DSP delivery in rats with a single dosing. PLA DSP-NP successfully reversed early signs of corneal rejection, leading to rat corneal graft survival for at least 6 months. Efficacious PLA DSP-NP doses did not affect IOP and showed no signs of ocular toxicity in rats for up to 6 months. Subconjunctival injection of DSP-NP is a promising approach for safely preventing and treating corneal graft rejection with the potential for improved patient adherence.

## Graphical abstract

**Figure:**
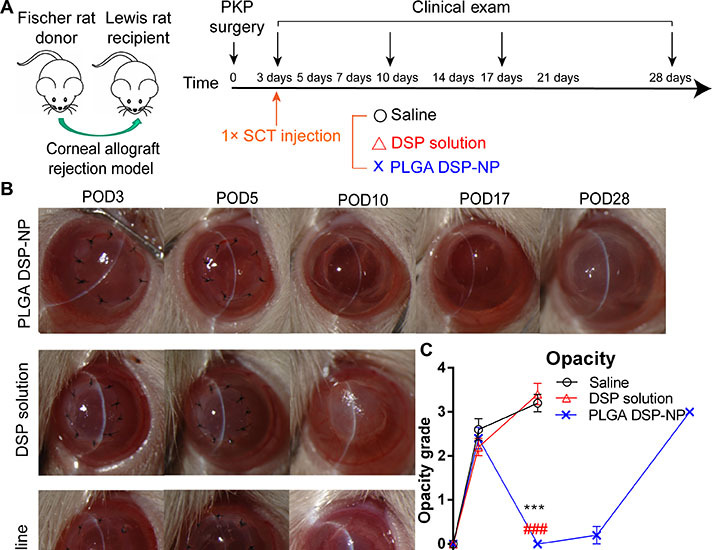

## Teaser

One single dose of DSP-NP achieves 6-month efficacy for treating corneal transplantation rejection.

## INTRODUCTION

Corneal disease is one of the leading causes of blindness and vision loss, affecting 4.2 million people worldwide (*1*). Corneal transplantation is the last step for visual rehabilitation once the disease severely affects corneal clarity (*2*). Each year, there are more than 60,000 corneal transplantations in the United States, and more than 184,576 corneal transplantation surgeries are performed worldwide (*3*, *4*). Immunological rejection of the donor cornea, which occurs in 10 to 50% of patients, is the most common reason for graft failure (*5*). Once a graft has been rejected and a repeated transplant is performed, these high-risk cases have the highest rates of rejection at ~50% (*6*, *7*). Furthermore, because of the limited availability of donor cornea tissues suitable for transplantation, a treatment that can effectively prevent and even reverse early signs of graft rejection would benefit patients and reduce the burden on the health care system.

Topical corticosteroids are the mainstay of immunosuppressant therapy for preventing and treating corneal graft rejection (*8*). However, topical eye drops typically provide low ocular bioavailability and require repetitive dosing (*9*). The clinical standard for preventing corneal graft rejection following keratoplasty is to apply corticosteroid eye drops as frequently as four to five times a day for at least 1 month and then gradually taper down the frequency of dosing over a total of 6 to 12 months (*10*, *11*). To rescue grafts with early signs of rejection, corticosteroid eye drops have to be used as frequently as once per hour while awake together with ophthalmic ointment while asleep (*11*, *12*). These intensive regimens can increase the chance of corticosteroid-associated side effects, such as intraocular pressure (IOP) elevation, leading to glaucoma and cataract (*13*, *14*). Lack of compliance with these intensive regimens is also a concern. Thus, there is a compelling need for a formulation that provides sustained release of corticosteroids to the anterior chamber for treating corneal graft rejection.

The water-soluble prodrug of dexamethasone (DEX), dexamethasone sodium phosphate (DSP), has been observed to accumulate in the anterior chamber after subconjunctival (SCT) injection, presumably due to transscleral diffusion (*15*), and thus is commonly administered at the end of corneal transplant surgery (*16*). Unfortunately, DSP is cleared rapidly from the eye within 24 hours after SCT injection, limiting the duration of effect and necessitating the prolonged use of topical eye drops (*11*). Previously, we developed poly(lactic-*co*-glycolic acid) (PLGA)–based DSP-loaded NPs (PLGA DSP-NP) that through a noncovalent cationic zinc bridging method with the terminal carboxylic acid group on the PLGA, sustained the release of DSP for about 2 weeks in vitro (*17*). Once weekly SCT injections of the PLGA DSP-NP formulation prevented corneal allograft rejection over a total of 2 months in rats (*17*). However, weekly SCT injections would be impractical clinically, necessitating the development of a long-lasting formulation.

Here, we describe the development of long-lasting DSP-NP using custom-synthesized dicarboxyl-terminated PLA polymers (PLA-2COOH) to extend the duration of DSP release while maintaining high DSP loading. We found that the PLA DSP-NP provided sustained drug concentrations in rat eyes for up to 6 months after a single SCT injection. We further demonstrate that a single SCT administration of PLA DSP-NP prevented corneal allograft rejection for up to 6 months in rats. Moreover, a single SCT of the PLA DSP-NP rescued corneal grafts with early signs of rejection and maintained graft survival for up to 6 months. In healthy rats, SCT injection of PLA DSP-NP caused no signs of ocular toxicity, as measured by IOP, corneal histology, and retinal function for up to 6 months. These results highlight the potential for a long-lasting PLA DSP-NP platform to replace the need for high-frequency topical corticosteroid eye drop administration for both the prevention and reversal of corneal graft rejection.

## RESULTS

### SCT injection of PLGA DSP-NP reversed the early signs of corneal allograft rejection and prolonged graft survival to 21 days

**Fig. 1.**
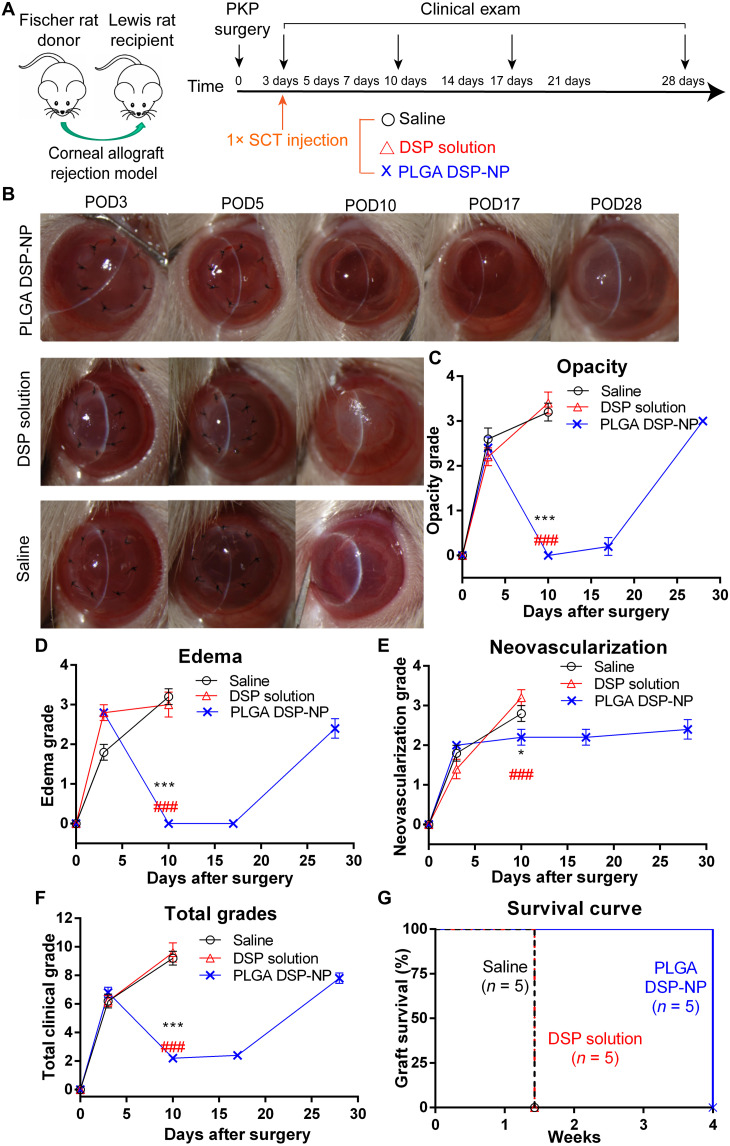
A single SCT injection of PLGA DSP-NP at POD3 provided short-term reversal of corneal graft rejection. (**A**) Treatment scheme of rat corneal allograft rejection. At POD3, a single 10-μl SCT injection of saline, DSP solution (DSP, 10 mg/ml), or PLGA DSP-NP (DSP, 10 mg/ml) was administered (*n* = 5 per group). Routine clinical evaluation was conducted at POD3, POD10, POD17, and POD28. (**B**) Representative photos of grafts at POD3, POD10, POD17, and POD28. (**C**) Corneal opacity, (**D**) edema, (**E**) neovascularization, (**F**) total clinical grade, and (**G**) graft survival rate. Data are shown as means ± SEM. For (C) to (F), statistical analysis at POD10 was calculated using one-way ANOVA with a Tukey’s post hoc test for multiple comparisons (PLGA DSP-NP compared with Saline: *P ≤ 0.05, **P ≤ 0.01, and ***P ≤ 0.001; PLGA DSP-NP compared with DSP solution: #*P*≤ 0.05, ##*P*≤ 0.01, ###*P*≤ 0.001). PKP, penetrating keratoplasty.

We previously demonstrated that our PLGA DSP-NP provided sustained drug concentrations in rat eyes for ~1 week following SCT injection (*18*) and prevented corneal graft rejection with weekly injection in a rat model (*17*). Here, we sought to investigate whether the PLGA DSP-NP could reverse early signs of rejection in rats. Conventional penetrating keratoplasty (PKP) was performed on inbred rats with different genetic backgrounds as previously described (*17*), and treatment was withheld until postoperative (PO) day 3 (POD3) (Fig. 1A). Early signs of rejection observed by slit lamp (Fig. 1B) and scoring of opacity (Fig. 1C), edema (Fig. 1D), and corneal neovascularization (CNV) (Fig. 1E) were confirmed on POD3, at which time rats received a single SCT injection of saline, DSP solution, or the PLGA DSP-NP (100 μg of DSP). Two days later (POD5), the grafts treated with saline and DSP solution exhibited increased opacity and edema (Fig. 1B) and, by total clinical grade (Fig. 1F), were completely rejected by POD10 (0% survival rate) (Fig. 1G). In contrast, treatment with PLGA DSP-NP led to gradually improved corneal transparency and reduced corneal edema from POD5 to POD10 (Fig. 1B). However, the corneal opacity and edema began to return past POD17, and all corneas in the PLGA DSP-NP–treated group were rejected by POD28 (Fig. 1G).

**Fig. 2.**
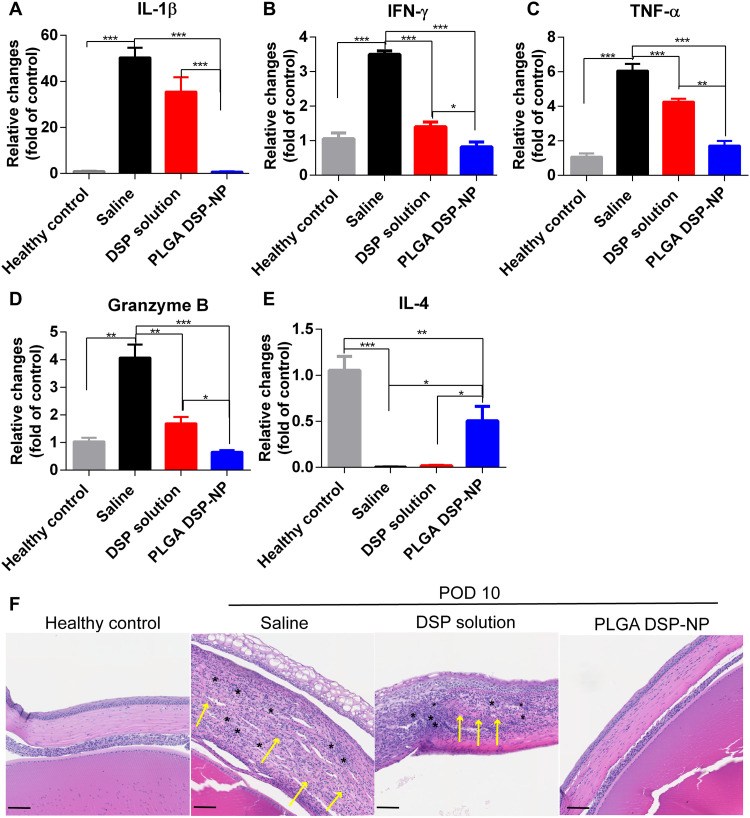
Reversal of corneal graft rejection with PLGA DSP-NP treatment is associated with down-regulation of pro-inflammatory genes and up-regulation of anti-inflammatory marker at POD10. Relative expression of (**A**) IL-1β, (**B**) IFN-γ, (**C**) TNF-α, (**D**) Granzyme B, and (**E**) IL-4 in cornea tissues at POD10 compared to healthy corneas (*n* = 6). Gene expression was normalized to glyceraldehyde-3-phosphate dehydrogenase (GAPDH) and analyzed using ΔΔ*C*T method. Results are presented as expression relative to that of healthy cornea tissue. (**F**) Histology images of grafts at POD10. Scale bars, 100 μm. Black asterisks indicate inflammatory cells. Yellow arrows indicate neovascularization in the cornea samples. Statistical significance was calculated by one-way analysis of variance (ANOVA) with Tukey’s multiple comparison tests (*P < 0.05, **P < 0.01, and ***P < 0.001). Data are shown as means ± SEM.

Because corticosteroids mainly affect cytokines secretion by lymphocytes and macrophages (*19*), we further analyzed inflammatory genes expression in the corneal grafts at POD10. Compared with DSP solution, the PLGA DSP-NP significantly down-regulated expression of pro-inflammatory interleukin-1β (IL-1β) (Fig. 2A), interferon-γ (IFN-γ) (Fig. 2B), tumor necrosis factor–α (TNF-α) (Fig. 2C), and Granzyme B (Fig. 2D), a cytolytic protein that promotes cell apoptosis and corneal immunological rejection (*20*). PLGA DSP-NP treatment also significantly up-regulated anti-inflammatory gene IL-4 expression (Fig. 2E). DSP solution treatment showed similar trends; however, the impact was significantly less than the DSP-NP treatment. Corneal histology at POD10 showed that the saline-treated corneal grafts had inflammatory cell infiltration (black asterisks), neovascularization (yellow arrows), and severe edema accompanied by thickening of the cornea (Fig. 2F). While not as severe as untreated animals, the corneal grafts of animals treated with DSP solution also had inflammatory cell infiltration, neovascularization, and edema (Fig. 2F). Notably, no substantial inflammatory cells and edema was found in the PLGA DSP-NP–treated corneal grafts (Fig. 2F). Rat corneal grafts treated with the PLGA DSP-NP on POD5 instead of POD3 showed complete graft rejection by POD12, highlighting the importance of diagnosing and treating early signs of rejection as early as possible (fig. S1).

### Long-lasting PLA DSP-NP demonstrated high DSP loading with sustained drug release in vitro up to 3 months

**Fig. 3.**
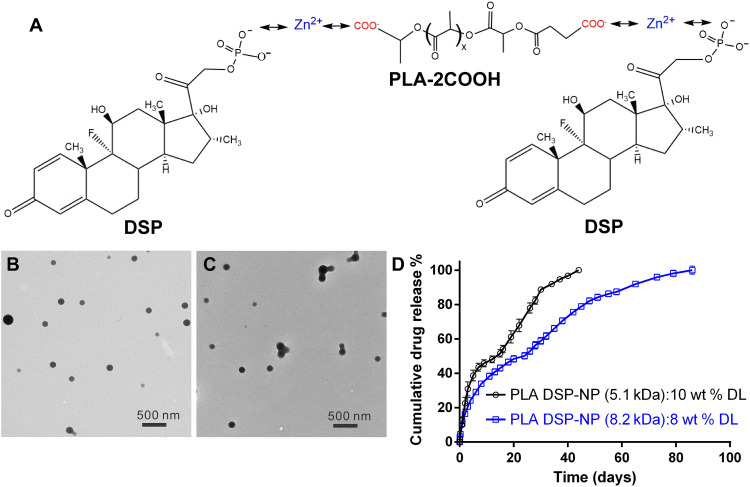
Design and characterization of PLA DSP-NP formulations. (**A**) DSP was loaded into PLA-2COOH NPs via zinc ion bridging between the carboxyl groups from PLA-2COOH and phosphate groups from DSP. Transmission electron microscope images of (**B**) PLA DSP-NP (5.1 kDa) and (**C**) PLA DSP-NP (8.2 kDa). (**D**) In vitro drug release profiles of PLA DSP-NP (5.1 kDa) and PLA DSP-NP (8.2 kDa) under sink conditions at 37°C. DL, DSP drug loading.

Water-soluble DSP has increased ocular bioavailability compared to low solubility DEX after SCT injection, but water-soluble drugs are more challenging to load for sustained drug release (*18*, *21*). One approach for loading DSP into biodegradable particles is to use a cationic zinc bridging between DSP and the carboxyl terminal groups on a biodegradable polymer such as PLGA, although there was a trade-off between polymer molecular weight/duration of release and the drug loading (*17*, *22*, *23*). To develop a longer-lasting DSP-NP formulation with similarly high drug loading as PLGA DSP-NP (*18*), we custom-synthesized dicarboxyl-terminated PLA polymers with weight averaged molecular weight (*M*_w_) of either 5.1 or 8.2 kDa (fig. S2). We introduced carboxyl terminal groups at both ends of low molecular weight PLA polymers to increase the DSP loading in the NP (Fig. 3A). In addition, the increased hydrophobicity of the PLA polymer would further slow the polymer degradation and prolong the drug release both in vitro and in vivo. PLA-2COOH polymers were characterized by ^1^H nuclear magnetic resonance and gel permeation chromatography (GPC) (fig. S2, A and B, and table S1). GPC analysis demonstrated that both polymers have the *M*_w_ of 5105 (5.1 kDa) and 8200 (8.2 kDa) with polydispersity index (PDI) of 1.36 and 1.25, respectively (table S1). Potentiometric titration was conducted to quantify the carboxyl content in the polymer. PLA-2COOH (5.1 and 8.2 kDa) contained carboxyl groups of 1297 ± 33 and 365 ± 6 μmol/g, respectively (fig. S3).

Both PLA-2COOH polymers were formulated into DSP-NP, namely, as PLA DSP-NP (5.1 kDa) and PLA DSP-NP (8.2 kDa), using the zinc ion bridging method as previously described (*17*). The PLA DSP-NPs were spherical in shape (Fig. 3, B and C) with particle sizes around 200 nm (Table 1). Pluronic F127 provided dense surface polyethylene glycol coating on the NP surface (*24*), as demonstrated by the near-neutral surface charge (ζ potential around −3 mV) (Table 1). Because of the increased carboxyl content of both polymers, the resulting drug loading of the PLA DSP-NP (5.1 kDa) and PLA DSP-NP (8.2 kDa) was 10% (w/w) and 8% (w/w), respectively (Table 1). Both PLA DSP-NP formulations demonstrated a longer duration of drug release than the PLGA-based formulation (*18*) without apparent burst release (<2% DSP release within the first 2 hours; Fig. 3D). The PLA DSP-NP (5.1 kDa) achieved sustained drug release for >30 days in vitro, and the PLA DSP-NP (8.2 kDa) provided even longer drug release for >3 months. The PLA DSP-NP (8.2 kDa) was selected for all animal studies moving forward and is henceforth referred to as PLA DSP-NP.

**Table 1.** Characterization of PLA DSP-NP formulations.

Formulation	Size (nm)	PDI	ζ potential (mV)	Drug loading (w/w)
PLA DSP-NP (5.1 kDa)	210.3 ± 9.1	0.06 ± 0.02	−3.0 ± 0.1	10%
PLA DSP-NP (8.2 kDa)	231.4 ± 7.1	0.16 ± 0.08	−3.6 ± 0.2	8%

### SCT injection of PLA DSP-NP provided sustained drug levels in the rat eye for 6 months

**Fig. 4.**
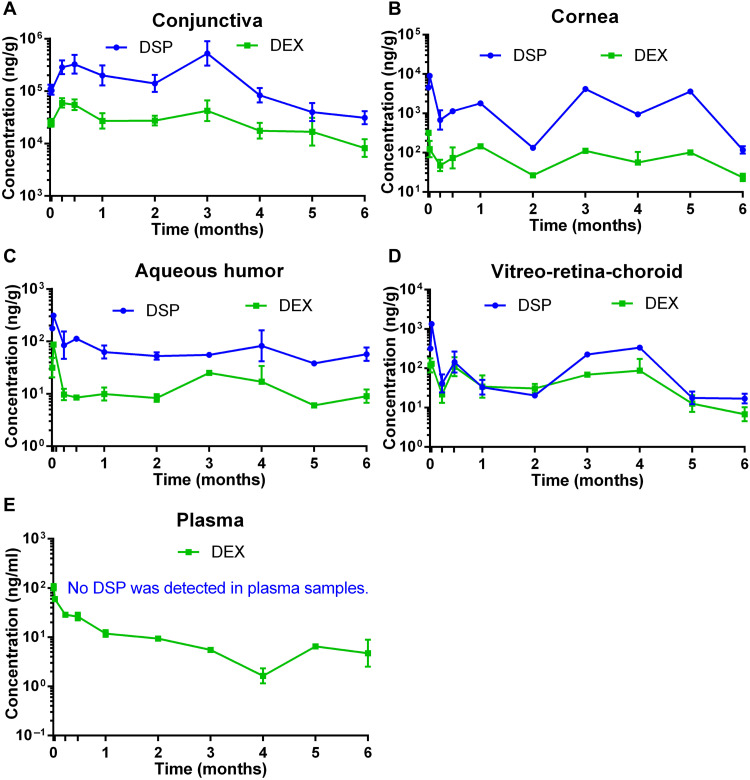
In vivo pharmacokinetic profiles of DSP and DEX after 30-μl SCT injection of PLA DSP-NP (600 μg of DSP) in rats. DSP and DEX concentrations in (**A**) conjunctiva, (**B**) cornea, (**C**) aqueous humor, (**D**) vitreo-retina-choroid, and (**E**) plasma at 6 hours, 1 day, 1 week, 2 weeks, 1 month, 2 months, 3 months, 4 months, 5 months, and 6 months after SCT injection of DSP-NP. Data are represented as means ± SEM. *n* = 5 to 6 eyes or *n* = 3 plasma samples.

It was previously demonstrated that the duration of drug release after periocular NP injection may be longer in vivo than the in vitro drug release under sink conditions (*25*). Here, we found that a single SCT injection of PLA DSP-NP was able to deliver sustained drug levels of DSP and parent drug DEX in the conjunctiva (Fig. 4A), cornea (Fig. 4B), aqueous humor (Fig. 4C), and vitreo-retina-choroid (Fig. 4D), with minimal systemic plasma exposure (Fig. 4E) for at least 6 months. In contrast, in our previous study, free DSP solution was rapidly cleared from rat eyes within a day after SCT injection (*17*).

The continuously high drug levels in conjunctiva tissue up to 6 months confirmed a long drug retention from the SCT injection of PLA DSP-NP (Fig. 4A). For other ocular tissues, a peak in DSP concentration was detected during the first month after the NP injection, followed by sustained DSP/DEX release afterward. Among all the ocular tissues, the highest drug concentration was observed in the conjunctiva, followed by the cornea, the vitreo-retina-choroid, and the aqueous humor (*26*, *27*). In plasma, the highest DEX concentration was detected at 6 hours after SCT injection of DSP-NP and gradually declined at later time points (Fig. 4E). There was no detectable DSP in the plasma (Fig. 4E), likely due to limited systemic absorption and rapid hydrolysis to parent DEX (*28*). AUC_0–180 days_ was calculated using noncompartmental analysis (fig. S4). In all the ocular tissues, the highest area under the curve (AUC) for DSP and DEX was observed in the conjunctiva, followed by the cornea, the vitreo-retina-choroid, and the aqueous humor. Drug levels were far lower in the plasma than in ocular tissues, highlighting the benefit of local, sustained release from a safety perspective.

### A single SCT injection of PLA DSP-NP successfully prevented corneal graft rejection for 6 months in rats

**Fig. 5.**
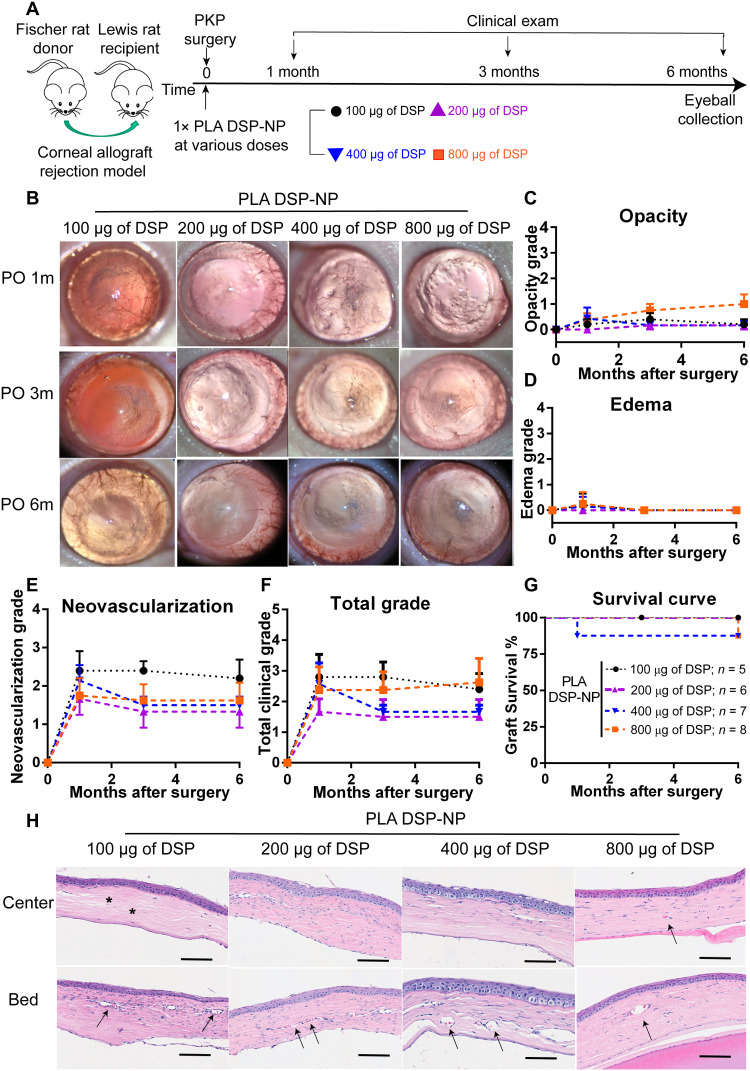
A single SCT injection of PLA DSP-NP prevented corneal allograft rejection. (**A**) Schedule for the efficacy study of PLA DSP-NP in preventing corneal allograft rejection. (**B**) Representative images of grafts at PO 1m, PO 3m, and PO 6m. (**C**) Corneal opacity grade, (**D**) edema grade, (**E**) neovascularization grade, (**F**) total clinical grades, and (**G**) graft survival rate. (**H**) Representative histology images of center (top row) and bed (bottom row) area of the cornea at PO 6m. Black asterisks represent the corneal stroma with a lack of corneal keratocytes. Black arrows indicate neovascularization in the corneal samples. Data are represented as means ± SEM. For (C) to (G), statistical analysis at each time points were calculated using one-way ANOVA with a Tukey’s post hoc test with multiple comparisons.

We then studied the efficacy of a single SCT injection of PLA DSP-NP for preventing the corneal graft rejection in rats. The schedule of the 6-month prevention study is shown in Fig. 5A. We first evaluated the efficacy of PLA DSP-NP at the dose containing 800 μg of DSP. As shown in Fig. 5B, at the dose of 800 μg of DSP, PLA DSP-NP maintained corneal transparency and prevented corneal allograft rejection for 6 months. As corticosteroid sparing is a common clinical strategy to avoid off-target side effects (*9*, *29*), we further investigated the efficacy of PLA DSP-NP at lower doses. As the doses were reduced to 400, 200, and 100 μg of DSP, PLA DSP-NP still maintained corneal transparency and prevented rat cornea allograft rejection for 6 months (Fig. 5B). Clinical evaluation also demonstrated that all grafts in the four groups were clear (average opacity grades of <1; Fig. 5C) with minimal edema (Fig. 5D) over the 6 months. All the groups exhibited average CNV grades around 2 over 6 months, which can be explained by the incomplete suppression of CNV provided by corticosteroids (*30*). Although the 100-μg dose provided relatively higher CNV grades during the 6-month treatment period, there was no statistical difference among all the groups at later time points (Fig. 5E). The minimum opacity grade (<1; Fig. 5C) and total clinical grades (average of <2.5; Fig. 5F) reflected a graft survival rate of >87% among all groups (Fig. 5G). There were only two animals with graft rejection; one of eight in the 400-μg group failed at postoperative (PO) 1 month (PO 1m), and one of eight in the 800-μg group failed at PO 6m (Fig. 5G).

Overall, the corneal histology at PO 6m showed relatively normal stroma, epithelium, and endothelium layer integrity, and a lack of corneal thickening or immune cell infiltration was observed (Fig. 5H). However, in the 100-μg dose group, corneal scarring was detected, as evidenced by the lack of keratocytes in the center part of the cornea stroma (black asterisks, Fig. 5H). Neovascularization was observed in the corneal bed area with minimal neovascularization in the center part of 800 μg of DSP-treated corneal graft (black arrows, Fig. 5H).

### A single SCT injection of PLA DSP-NP effectively reversed early signs of corneal graft rejection and maintained the graft survival for 6 months

**Fig. 6.**
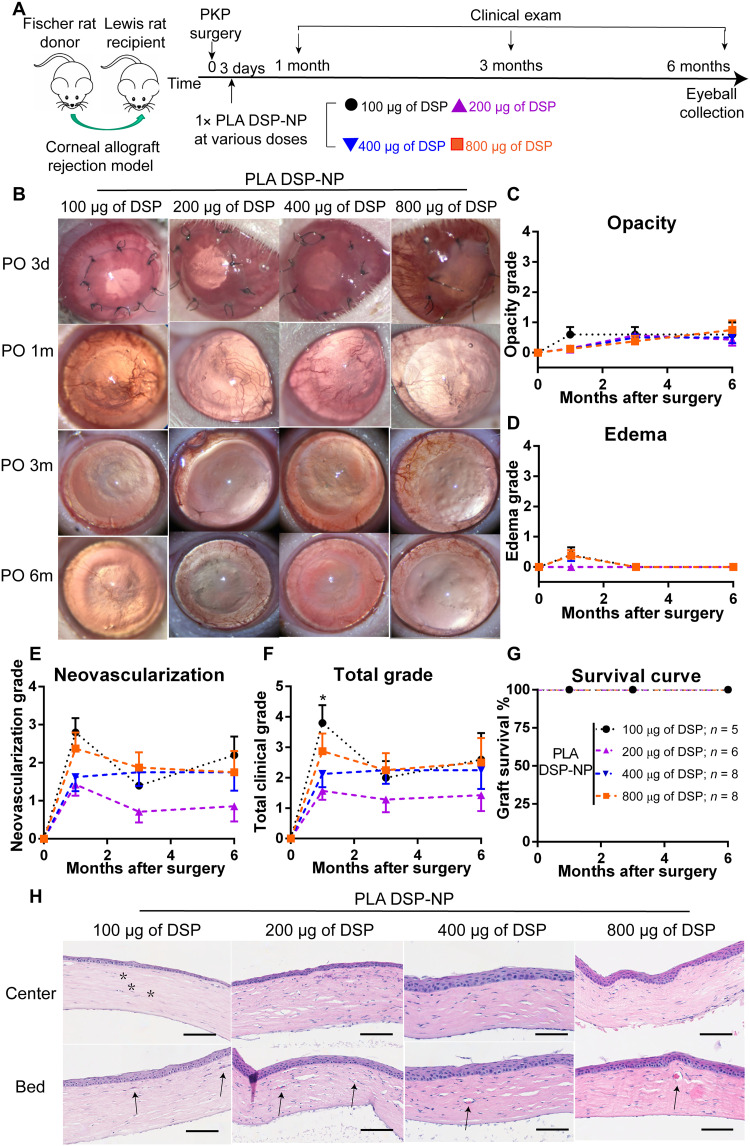
A single SCT injection of PLA DSP-NP rescued corneal allografts with signs of early injection and maintained graft survival up to 6 months. (**A**) Schedule for the efficacy study of PLA DSP-NP in reversing corneal allograft with signs of early rejection. (**B**) Representative images of grafts at PO 1 month, 3 months, and 6 months. (**C**) Corneal opacity, (**D**) edema, and (**E**) neovascularization, (**F**) total clinical score, and (**G**) graft survival curves. (**H**) Representative histology images of center (top row) and bed (bottom row) area of the cornea at PO 6m. Black asterisks represent the corneal stroma with a lack of corneal keratocytes. Black arrows indicate neovascularization in the corneal samples. Data are represented as means ± SEM. For (C) to (G), statistical analysis at each time point was calculated using one-way ANOVA with a Tukey’s post hoc test for multiple comparisons. (*P ≤ 0.05 for 100 to 200 μg.)

To assess the potential for the long-lasting PLA DSP-NP formulation to reverse early signs of corneal graft rejection, various doses of PLA DSP-NP were injected SCT on POD3 (Fig. 6A). Similarly, we started an efficacy study at the high dose of PLA DSP-NP containing 800 μg of DSP. As shown in Fig. 6B, PLA DSP-NP (800 μg of DSP) successfully rescued grafts with early signs of rejection, resulting in clear grafts at PO 1m and up to 6 months. Then, we further reduced the dose of PLA DSP-NP to 400, 200, and 100 μg of DSP. Encouragingly, all doses of PLA DSP-NP successfully rescued grafts with early signs of rejection, resulting in clear grafts at PO 1m and up to 6 months (Fig. 6B). Animals treated with PLA DSP-NP had low average opacity grades (<1; Fig. 6C) and edema grades (<0.5; Fig. 6D). CNV was detected in all dose groups with an average score of <3 (Fig. 6E). All groups presented total clinical scores <4 (Fig. 6F), consistent with 100% graft survival in all groups (Fig. 6G).

The corneal histology at PO 6m presented relatively normal structure integrity, and a lack of corneal thickening or immune cell infiltration was observed (Fig. 6H). However, neovascularization was detected in the recipient area of all the grafts (black arrows, Fig. 6H). Similar to that shown in Fig. 5H, grafts of rats treated with 100 μg of DSP had a reduced number of keratocytes in the stroma area, suggesting reduced efficacy compared to higher doses (black asterisks, Fig. 6H). All the corneal grafts presented similar compact structure and structural integrity as the healthy corneas, with no apparent inflammatory cell infiltration or abnormal blood vessels (Fig. 6H).

### Safety evaluation after a single SCT injection of PLA DSP-NP was followed for 6 months

**Fig. 7.**
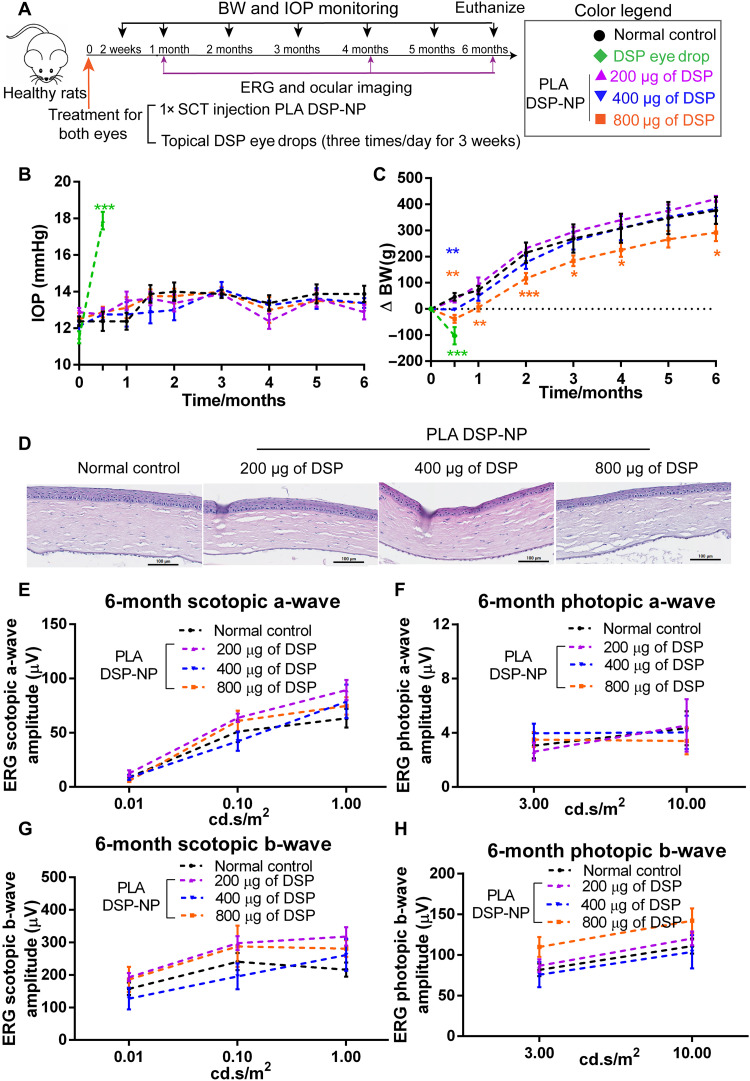
Safety evaluation after a single SCT injection of PLA DSP-NP. (**A**) Schedule of safety study. (**B**) Rat IOP and (**C**) change in body weight (BW) (%) after single SCT injection of PLA DSP-NP (200, 400, or 800 μg of DSP). Topical 0.1% DSP eye drops (three times/day for 3 weeks) were applied as positive control, and untreated rats were used as negative control. (**D**) Representative hematoxylin and eosin staining of cornea collected at 6 months after PLA DSP-NP injection. Quantification of the amplitude of (**E**) scotopic a-wave, (**F**) photopic a-wave, (**G**) scotopic b-wave, and (**H**) photopic b-wave at 6 months. *N* = 4 independent animals per group were used for body weight monitoring. *N* = 6 to 8 independent eyes per group for IOP and ERG studies. Data are represented as means ± SEM. Statistical analysis for (B) and (C) and (E) to (H), two-way ANOVA with multiple comparisons was used. (* for comparison with healthy control: *P ≤ 0.05, **P ≤ 0.01, and ***P ≤ 0.001.)

It has been reported that the long-term corticosteroid administration is associated with increased IOP and body weight changes (*31*–*34*). Thus, we next conducted a 6-month safety study in healthy rats to monitor ocular IOP and body weight after a single SCT injection of PLA DSP-NP (200, 400, and 800 μg of DSP) in both eyes compared to three times daily of 0.1% DSP topical eye drops for 3 weeks (Fig. 7A). While a significant increase in IOP was observed with the three times daily eye drops at the second week after dosing, there was no change in IOP with all doses of PLA DSP-NP through the full 6 months (Fig. 7B). The topical eye drops further were associated with a significant decrease in body weight of ~29% at the second week (Fig. 7C), which further increased to a loss of ~45% of body weight by 3 weeks, necessitating cessation of treatment. While only 400- and 800-μg doses of PLA DSP-NP treatment induced a dose-dependent reduction in body weight at the second week after SCT injection, the body weight recovered and steadily increased in all groups (Fig. 7C). Although the body weight of rats receiving the 800-μg dose recovered and increased throughout the 6 months, their body weight remained significantly lower than the rats receiving the 200- and 400-μg doses (Fig. 7C).

Another concern regarding the long-term usage of corticosteroid is cataract (*9*). As shown in fig. S5, all the eyes were clear with no signs of cataract over 6 months. Similarly, no obvious histology change was observed in rat corneas at 6 months after PLA DSP-NP administration (Fig. 7D). Furthermore, compared with untreated control rats, electroretinogram (ERG) on rats receiving PLA DSP-NP treatment were indistinguishable (Fig. 7, E to H, and fig. S6).

## DISCUSSION

Despite advances in surgical technique for corneal transplantations (*35*), immunological graft rejection is still a major cause of graft failure and need for repeated procedures (*6*, *7*). Because corneal rejection begins to develop immediately after corneal graft transplantation (*36*), surgeons typically administer a SCT injection of DSP at the end of surgery in addition to prescribing corticosteroid drops to be used for 6 to 12 months after surgery (*12*, *16*). While the ocular bioavailability of water-soluble DSP after SCT injection is relatively high compared to poorly soluble DEX, drug retention time in the eye is typically less than 1 day (*15*). The poorly soluble DEX has been formulated into topical suspensions while it has a short half-life of less than 2 hours (*37*). Because of the limited intraocular absorption and rapid clearance of topical eye drops, repetitive dosing is required, compromising patient compliance and increasing likelihood of corticosteroid-related side effects (*38*, *39*). Particularly in the case where early signs of rejection are evident, hourly eye drop dosing is onerous, and the need for subsequent transplantation procedures further increases risk of graft rejection (*40*).

The use and limitations of DSP in the clinical management of corneal transplantation procedures inspired us to develop sustained-release formulations for SCT injection, although achieving loading and sustained release of highly water-soluble drugs such as DSP is challenging. Previously, we and other researchers described the development of water-soluble corticosteroid-loaded NPs using zinc cations to bridge the phosphate group on the corticosteroid to terminal carboxyl groups on PLGA polymers (*17*, *18*, *22*, *23*). As DSP is highly water soluble (water solubility of >50 mg/ml), this bridging interaction is key in achieving sufficient drug loading and sustained drug release. While DSP-NPs prepared with conventional ester-terminated PLGA polymer contained only a fraction of a percent of DSP by weight (<0.2 wt % DSP loading), DSP-NPs composed of carboxyl-terminated PLGA (3.2 kDa) had ~8% (w/w) DSP loading and provided DSP release for at least 1 week in vitro (*17*). The duration of in vitro drug release could be extended to 3 months using a higher molecular weight carboxyl-terminated PLGA (32 kDa), although the DSP loading was only ~1% (w/w), indicating that the hydrophobic interaction was not the determinant of high DSP loading in the NP and highlighting the importance of the carboxyl groups of polymers instead (*18*). The DSP-NP composed of PLGA (3.2 kDa) provided sustained DSP levels in the rat eye for about 1 week, and we demonstrated that this led to effective prevention of corneal allograft rejection in rats with once weekly dosing (*17*). While more effective than free DSP injection and safer than daily eye drops (*17*), once weekly is not a clinically feasible injection frequency. Although the duration of action of the first-generation PLGA DSP-NP would not be feasible as a long-term strategy to replace eye drops for prevention of corneal graft rejection, the fast-acting release that lasts for about a week could be a feasible option for replacing intensive eye drops to reverse the early signs of graft rejection. Encouragingly, we observed here that a single SCT injection of the PLGA DSP-NP on POD3 was effective in reversing the early signs of graft rejection, while free DSP injection was unable to rescue the grafts from failure. PLGA DSP-NP injection also extended the duration of the graft survival to POD17, but all corneal grafts were rejected by POD28. Although promising, this case still necessitates the development of a long-lasting formulation.

Here, we designed dicarboxyl-terminated PLA polymers to formulate long-lasting DSP-NP that provided sustained corticosteroid levels in the rat eye for at least 6 months with a single SCT injection. We custom-synthesized dicarboxyl-terminated PLA polymers, which would both increase the number of available carboxyl groups and the polymer hydrophobicity allowing for the use of lower molecular weight PLA while maintaining a slower degradation rate than PLGA. Our PLA DSP-NP that composed of PLA-2COOH (8.2 kDa) had 8% (w/w) DSP loading and released DSP for at least 3 months in vitro. However, upon evaluating intraocular drug levels after a single SCT injection in rats, drug concentrations were sustained in ocular compartments for at least 6 months. This is consistent with prior observations that the duration of in vitro drug release may not directly correlate with the duration of in vivo drug release upon ocular injection (*25*, *41*). The longer drug release in vivo over in vitro can be explained by the extended NP retention and delayed NP clearance at the injection site. Even at 6 months after the PLA DSP-NP injection, we were able to observe particles at the injection site liquid chromatography with tandem mass spectrometry (LC-MS/MS) analysis also demonstrated remaining DSP concentration of 31 μg/g in the conjunctival tissues at 6 months after the administration of PLA DSP-NP.

SCT injection has a long history of use for achieving drug delivery to the anterior segment of the eye, although SCT injection of free drug solutions is often associated with rapid systemic exposure and limited ocular bioavailability (*42*). In contrast, our PLA DSP-NP provided sustained DSP concentrations in the rat cornea and aqueous humor for 6 months. In addition, the ocular exposure (AUC_0–180 days_) of DSP in the anterior segment of the eye was around 200-fold higher than that in the systemic circulation after PLA DSP-NP injection. In the efficacy study, we first demonstrated that a dose of 800 μg of PLA DSP-NP was effective in preventing and reversing corneal graft rejection while maintaining corneal graft survival out of 6 months. Encouragingly, even lower doses of 400, 200, and even 100 μg of DSP-NP were still effective in preventing and reversing the corneal graft rejection and maintained the graft survival for 6 months, with few exceptions. Moreover, there was no significant difference in the overall efficacy of PLA DSP-NP among all the doses. Furthermore, in the safety study, no significant IOP increase was detected over 6 months after the SCT injection of PLA DSP-NP at all doses level. Although the highest dose of PLA DSP-NP caused a slight acute decrease in body weight, the impact was less than the repeated corticosteroid eye drops dosing regimen (three times/day for 3 weeks). The lower doses of PLA DSP-NP had no effect on body weight. Using the minimal possible effective dose would be of particular benefit to pediatric patients (*43*) and patients with pre-existing ocular diseases including glaucoma, history of ocular trauma that requiring extra cautious usage of topical corticosteroids (*14*, *44*). Identifying the optimal minimum dose of PLA DSP-NP to maximize safety and efficacy while also considering dose scaling to larger eyes will be the subject of future studies.

Despite the encouraging efficacy and safety demonstrated for PLA DSP-NP, some aspects can be further improved. First, in the efficacy studies, rats receiving all doses of PLA DSP-NP still demonstrated low levels of CNV. Corticosteroids have previously been shown to only partially suppress CNV and have limited efficacy in reducing existing CNV (*30*, *45*). It is possible that more complete control of CNV after PKP surgery could be achieved using concomitant anti-angiogenic treatment. For example, tyrosine kinase inhibitors and anti–vascular endothelial growth factor therapies have been shown to be effective in reducing CNV preclinically (*46*, *47*). Second, considering the large size differences between human and rodent eyes, ocular pharmacokinetic and safety studies on larger animals should be conducted to facilitate future clinical translation. Because of heightened ocular sensitivity and eye size that approaches that of humans, rabbits are the most commonly used species for ocular research (*48*). Rabbits have been used in corneal transplantation studies; however, the rabbit corneal endothelial cells can proliferate in vivo and would tend to heal spontaneously (*49*), and the corneal grafts incline to survive indefinitely even without the use of immunosuppressants when allograft placed on nonvascularized rabbit graft beds (*50*). Therefore, the rabbits may not be a good model in our study aiming to evaluate the efficacy of PLA DSP-NP to prevent and treat normal risk corneal allograft rejection. To achieve the corneal transplantation rejection, some researchers induced neovascularization on rabbit corneas to make the corneal transplant as high-risk corneal transplantation, which is a different animal disease model (*50*). On the contrary, the rat eyes are big enough to do the corneal transplantation with chronic immunological rejection when a corneal graft from an inbred strain (e.g. Fischer rats) was implanted to a different inbred strain (e.g. Lewis rats). However, rabbit has the sclera thickness about the half of the human sclera thickness (0.4 ~ 0.9 mm), while sclera thickness in rat is much thinner (<0.1 mm) (*51*, *52*). The thicker sclera in larger animals may lead to slower drug diffusion into the eye after the SCT injection. In addition, rat eye has much slower aqueous humor flow rate of 0.35 μl/min than that of rabbit eye, which shares similar aqueous humor flow with human eye (2.7 to 2.8 μl/min), and the higher aqueous humor flow rate may contribute to the faster drug clearance from the eye (*51*).

In summary, we developed a PLA DSP-NP that could improve patient compliance by eliminating the repeated application of topical corticosteroid eye drops, decreases the risk of corticosteroid induced side effects, and leads to the 6-month efficacies of preventing and treating corneal graft rejection. The DSP-NPs mainly composed of PLGA/PLA and zinc, which are widely used in the ophthalmic products (*53*, *54*), thus holding great potential for the clinical translation of this drug delivery platform. Given the broad applications of corticosteroids, the PLA DSP-NP should have broad potential clinical impact.

## MATERIALS AND METHODS

### Materials

PLGA (50:50, *M*_w_ ~ 3.2 kDa, single carboxyl-terminated) was purchased from Lakeshore Biomaterials (Evonik, Birmingham, AL). The dicarboxyl-terminated poly(d,l-lactide acid), PLA-2COOH (5.1 kDa), and PLA-2COOH (8.2 kDa) were custom-synthesized by Polymer Source Inc. (Quebec, Canada) (details were provided in the Supplementary Materials). DSP salt (0215756594) was purchased from MP Biomedicals (Santa Ana, CA). Pluronic F127 (a polyethylene oxide-polypropylene oxide-polyethylene oxide triblock copolymer), triethanolamine (TEOA; T58300), EDTA (324504) solution (0.5 M), zinc acetate dihydrate (383317), tetrahydrofuran (THF; T5267), and all other organic solvents were purchased from Sigma-Aldrich (St. Louis, MO).

### NP preparation

Both PLGA DSP-NP and PLA DSP-NP were prepared by nanoprecipitation method in the presence of F127, as previously described (*17*). DSP-zinc complex was first prepared by adding 1 ml of 0.5 M zinc acetate to a 0.5 ml of DSP aqueous solution containing 20 mg of DSP. After centrifuge at 20,000*g* for 5 min, the DSP-zinc precipitate was further dissolved in 0.5 ml of THF together with 100 mg of PLGA or PLA-2COOH polymers. The pH of the polymer solution was adjusted to 7 to 8 by TEOA to get a clear solution. Then, the solution was added dropwise into a 75 ml of 5% (w/v) Pluronic F127 aqueous solution under stirring to form DSP-NP. After complete removal of THF by solvent evaporation via a Buchi rotary evaporator (Buchi Corporation, DE, USA), 1 ml of 0.5 M EDTA aqueous solution was added to remove unencapsulated DSP-zinc complex. DSP-NPs were then collected by centrifugation at 8000*g*, washed with 5% F127, and resuspended in ultrapure water.

### Characterization of DSP-NP

A Zetasizer Nano ZS90 (Malvern Instruments, Southborough, MA) was used to determine the particle size, size distribution, and surface charge of DSP-NP. Transmission electron microscope (TEM) images of DSP-NP were obtained using a Hitachi H7600 TEM (Hitachi Co. Ltd., Tokyo, Japan). To measure drug loading, 50 μl of DSP-NP was lyophilized, weighed, and dissolved in 500 μl of acetonitrile. One microliter of 50 mM EDTA was added to solubilize DSP-zinc complex, and the DSP concentration was measured by high-performance liquid chromatography/ultraviolet (HPLC/UV) analysis.

HPLC analysis was performed on a Shimadzu Prominence LC system (Kyoto, Japan) equipped with a Pursuit 5 C18 column (Varian Inc., Lake Forest, CA). Isocratic separation was conducted using the mobile phases consisting of acetonitrile/water [35/65 (v/v)] containing 0.1% trifluoroacetic acid (flow rate = 1 ml/min). Column effluent was monitored by UV detection at 241 nm.

### In vitro drug release of DSP-NP

Four hundred microliters of DSP-NP suspension was sealed in a dialysis tubing cellulose membrane (*M*_w_ cutoff: 10 kDa; Sigma-Aldrich, St. Louis, MO). The sealed dialysis membrane was placed into a 50-ml conical tube containing 12 ml of release media [phosphate-buffered saline (PBS); pH 7.4] and incubated at 37°C on a platform shaker (140 rpm). The entire release medium was collected at predetermined intervals and replaced with another 12 ml of fresh PBS. DSP concentration in the collected release medium was measured by HPLC/UV analysis.

### Animals

All experimental protocols were approved by the Virginia Commonwealth University (VCU) Institutional Animal Care and Use Committee (IACUC). Six- to 8-week-old male Lewis, Fischer, and Sprague Dawley rats were obtained from Charles River Laboratories. Sprague Dawley rats were used for ocular pharmacokinetic study and safety study. For the corneal transplantation studies, Lewis rats were used as the receptor animals and Fisher rats were used as donor animals in the PKP studies. All experimental animals were cared by the VCU's Department of Animal Resources. Animals were anesthetized before euthanasia.

### Pharmacokinetic studies

Male Sprague Dawley rats (6 to 8 weeks old) were anesthetized by intramuscular injection of a mixture of ketamine (80 mg/kg) and xylazine (8 mg/kg). Each rat was given bilateral SCT injection of PLA DSP-NP suspension containing 600 μg of DSP. At the indicated time intervals, 6 hours, 1 day, 7 days, 14 days, 1 month, 2 months, 3 months, 4 months, 5 months, and 6 months after injection, the rats were euthanized and 500 μl of whole blood was collected through heart puncture and stored in BD Vacutainer blood collection tubes with EDTA coating (NJ, USA). The whole eyeballs were excised and dissected into the aqueous humor, cornea, and vitreo-retina-choroid, which were then placed in preweighted tubes. Plasma was collected by centrifuging whole blood samples at 1000*g* for 15 min. Collected tissue and plasma were stored at −80°C before LC-MS/MS analysis.

### Corneal transplantation surgery

Corneal allograft transplantation was performed as previously described with a donor cornea graft from male Fischer rats (6 to 8 weeks old) into the corneal bed of recipient male Lewis rats (6 to 8 weeks old) (*17*). Briefly, Fischer donor rats were euthanized, and the central corneal button of both eyes was removed with a 3.5-mm trephine and kept in physiological solution ready for use. PKPs were performed by an experienced corneal surgeon under an operating ophthalmic microscope (Zeiss, Germany). The cornea recipient Lewis rats were anesthetized, and the pupils were dilated by 0.5% tropicamide eye drops. Only one eye of the Lewis rats received corneal transplantation. The paracentesis was performed before trephinization, and the anterior chamber was filled with Healon GV (Johnson & Johnson, USA). The corneal buttons were removed from the receptor Lewis rats with the 3.0-mm trephine. The donor corneal buttons were sutured to receptor corneas with eight interrupted 10-0 nylon sutures.

### Clinical evaluation

In the case of graft infection, endophthalmitis, or cataract, rats were immediately euthanized. Three parameters were evaluated for the examination of the corneal grafts including corneal transparency, edema, and neovascularization. The clinical evaluations were done at blind manner. Detailed clinical scores are listed in table S2. Grafts were considered to have been rejected only when the total score reached 5 with an opacity score of ≥3, as previously described (*17*). Digital ocular microscopic images were taken under ophthalmic microscope using Labcam (ilabcam.com) with iPhone 8.

### Safety study

Healthy male Sprague Dawley rats (6 to 8 weeks old) were used for safety evaluation of PLA DSP-NP. Baseline body weight and IOP were first collected before SCT administration. Each rat was given bilateral SCT injection of PLA DSP-NP suspension containing 200, 400, or 800 μg of DSP. Rats receiving topical eye drops were bilaterally dosed with 0.1% DSP (Bausch + Lomb, Laval, Canada) three times/day (5 hours apart) for 3 weeks. Rats receiving no treatment were used as negative control. Body weight and IOP were evaluated every other week during the first month followed by monthly monitoring until 6 months after PLA DSP-NP injection. Noninvasive IOP measurements were conducted using an Icare Tonolab (Icare, Helsinki, Finland). The IOP of both eyes was measured. The IOP value for each eye was from the average of three measurements, and each measurement was from six consecutive readings. At 1, 4, and 6 months after PLA DSP-NP injection, ocular microscopic images were taken using Labcam (ilabcam.com) with iPhone 8 and retinal function was assessed using ERG as described in the Supplementary Materials.

### Histological examination

At the end of the studies, rats were euthanized and the eyes were enucleated. The whole eyeballs were fixed with 10% formalin for 24 hours before paraffin embedding. Axial sections (5 μm) with antero-posterior orientation (from the cornea to the optic nerve) were cut and stained with hematoxylin and eosin. The slides were analyzed and graded by an ophthalmic pathologist under masked conditions.

### Polymerase chain reaction analysis

PKP surgery was conducted on Lewis rats as described before. At POD3, 10 μl of saline, DSP solution, or PLGA DSP-NP (100 μg of DSP) was administered through SCT injection. At POD10, rats were euthanized and whole cornea was collected. Cornea was first cut into small pieces and further homogenized in 400 μl of TRIzol (Thermo Fisher Scientific, USA) using a Bullet Blender Storm (Next Advance, USA). After centrifugation, the supernatant was collected and extracted with 80 μl of chloroform followed by further extraction using isopropanol. Last, the RNA was precipitated and resuspended in water. After quantification, the complementary DNA (cDNA) was generated using a high-capacity cDNA reverse transcription kit (Applied Biosystems, USA). Polymerase chain reaction analysis was conducted in three replicates using SYBR Green Master Mix (Applied Biosystems, USA). Primer sequences can be found in table S1. The ∆∆*C*T method was used to analyze gene expression, normalized to reference gene glyceraldehyde-3-phosphate dehydrogenase.

### Statistical analysis

Statistical analysis for clinical scores evaluation was performed by either one-way analysis of variance (ANOVA) followed by Tukey’s post hoc test under multiple comparison (α = 0.05) or two-way ANOVA analysis followed by multiple comparison (IBM SPSS version 26, NY, USA). The Kaplan-Meier method was used to assess the graft survival rates. All figures were prepared using GraphPad Prism, and the data were displayed as mean ± SEM.

## Supplementary Materials

### This PDF file includes:

Supplementary Materials and Methods Figs. S1 to S6 Tables S1 to S3 References

## Notes

View/request a protocol for this paper from *Bio-protocol*.

## References

[1] World Health Organization, Blindness and vision impairment (2020);www.who.int/news-room/fact-sheets/detail/blindness-and-visual-impairment.

[2] D. T. H. Tan, J. K. G. Dart, E. J. Holland, S. Kinoshita, Corneal transplantation. Lancet 379, 1749–1761 (2012). 22559901 10.1016/S0140-6736(12)60437-1

[3] T. M. M. Ways, W. M. Lau, V. V. Khutoryanskiy, Chitosan and its derivatives for application in mucoadhesive drug delivery systems. Polymers 10, 267 (2018). 30966302 10.3390/polym10030267 PMC6414903

[4] P. Gain, R. Jullienne, Z. He, M. Aldossary, S. Acquart, F. Cognasse, G. Thuret, Global Survey of Corneal Transplantation and Eye Banking. JAMA Ophthalmol. 134, 167–173 (2016). 26633035 10.1001/jamaophthalmol.2015.4776

[5] J. Yin, Advances in corneal graft rejection. Curr. Opin. Ophthalmol. 32, 331–337 (2021). 33989234 10.1097/ICU.0000000000000767 PMC9290782

[6] Y. Qazi, P. Hamrah, Corneal allograft rejection: Immunopathogenesis to therapeutics. J. Clin. Cell Immunol. 2013(Suppl. 9), 006 (2013). 24634796 10.4172/2155-9899.S9-006 PMC3954811

[7] T. B. Abud, A. Di Zazzo, A. Kheirkhah, R. Dana, Systemic immunomodulatory strategies in high-risk corneal transplantation. J. Ophthalmic Vis. Res. 12, 81–92 (2017). 28299010 10.4103/2008-322X.200156 PMC5340067

[8] I. Ezon, C. Y. Shih, L. M. Rosen, T. Suthar, I. J. Udell, Immunologic graft rejection in descemet's stripping endothelial keratoplasty and penetrating keratoplasty for endothelial disease. Ophthalmology 120, 1360–1365 (2013). 23531352 10.1016/j.ophtha.2012.12.036

[9] S. A. Gaballa, U. B. Kompella, O. Elgarhy, A. M. Alqahtani, B. Pierscionek, R. G. Alany, H. Abdelkader, Corticosteroids in ophthalmology: Drug delivery innovations, pharmacology, clinical applications, and future perspectives. Drug. Deliv. Transl. Res. 11, 866–893 (2020). 10.1007/s13346-020-00843-z 32901367

[10] F. W. Price Jr.., D. A. Price, V. Ngakeng, M. O. Price, Survey of steroid usage patterns during and after low-risk penetrating keratoplasty. Cornea 28, 865–870 (2009). 19654531 10.1097/ICO.0b013e318197ef07

[11] E. Guilbert, J. Bullet, O. Sandali, E. Basli, L. Laroche, V. M. Borderie, Long-term rejection incidence and reversibility after penetrating and lamellar keratoplasty. Am. J. Ophthalmol. 155, 560–569.e2 (2013). 23218931 10.1016/j.ajo.2012.09.027

[12] W. J. Stark, M. G. Maguire; The Collaborative Corneal Transplantation Studies Research Group, Design and methods of the collaborative corneal transplantation studies. Cornea 12, 93–103 (1993). 8500329 10.1097/00003226-199303000-00001

[13] M. R. Razeghinejad, L. J. Katz, Steroid-induced iatrogenic glaucoma. Ophthalmic Res. 47, 66–80 (2011). 21757964 10.1159/000328630

[14] G. Roberti, F. Oddone, L. Agnifili, A. Katsanos, M. Michelessi, L. Mastropasqua, L. Quaranta, I. Riva, L. Tanga, G. Manni, Steroid-induced glaucoma: Epidemiology, pathophysiology, and clinical management. Surv. Ophthalmol. 65, 458–472 (2020). 32057761 10.1016/j.survophthal.2020.01.002

[15] O. Weijtens, E. J. Feron, R. C. Schoemaker, A. F. Cohen, E. G. W. M. Lentjes, F. P. H. T. M. Romijn, J. C. van Meurs, High concentration of dexamethasone in aqueous and vitreous after subconjunctival injection. Am. J. Ophthalmol. 128, 192–197 (1999). 10458175 10.1016/s0002-9394(99)00129-4

[16] J. B. Randleman, R. D. Stulting, Prevention and treatment of corneal graft rejection: Current practice patterns (2004). Cornea 25, 286–290 (2006). 16633028 10.1097/01.ico.0000178731.42187.46

[17] Q. Pan, Q. Xu, N. J. Boylan, N. W. Lamb, D. G. Emmert, J. C. Yang, L. Tang, T. Heflin, S. Alwadani, C. G. Eberhart, W. J. Stark, J. Hanes, Corticosteroid-loaded biodegradable nanoparticles for prevention of corneal allograft rejection in rats. J. Control. Release 201, 32–40 (2015). 25576786 10.1016/j.jconrel.2015.01.009 PMC6037178

[18] L. Luo, J. Yang, Y. Oh, M. J. Hartsock, S. Xia, Y.-C. Kim, Z. Ding, T. Meng, C. G. Eberhart, L. M. Ensign, J. E. Thorne, W. J. Stark, E. J. Duh, Q. Xu, J. Hanes, Controlled release of corticosteroid with biodegradable nanoparticles for treating experimental autoimmune uveitis. J. Control. Release 296, 68–80 (2019). 30660629 10.1016/j.jconrel.2019.01.018 PMC6476551

[19] V. S. Gorantla, J. H. Barker, J. W. Jones Jr., K. Prabhune, C. Maldonado, D. K. Granger, Immunosuppressive agents in transplantation: Mechanisms of action and current anti-rejection strategies. Microsurgery 20, 420–429 (2000). 11150994 10.1002/1098-2752(2000)20:8<420::aid-micr13>3.0.co;2-o

[20] R. I. Lechler, M. Sykes, A. W. Thomson, L. A. Turka, Organ transplantation—How much of the promise has been realized? Nat. Med. 11, 605–613 (2005). 15937473 10.1038/nm1251

[21] M. R. Prausnitz, J. S. Noonan, Permeability of cornea, sclera, and conjunctiva: A literature analysis for drug delivery to the eye. J. Pharm. Sci. 87, 1479–1488 (1998). 10189253 10.1021/js9802594

[22] T. Ishihara, M. Takahashi, M. Higaki, Y. Mizushima, Efficient encapsulation of a water-soluble corticosteroid in biodegradable nanoparticles. Int. J. Pharm. 365, 200–205 (2009). 18804157 10.1016/j.ijpharm.2008.08.030

[23] T. Ishihara, N. Izumo, M. Higaki, E. Shimada, T. Hagi, L. Mine, Y. Ogawa, Y. Mizushima, Role of zinc in formulation of PLGA/PLA nanoparticles encapsulating betamethasone phosphate and its release profile. J. Control. Release 105, 68–76 (2005). 15955367 10.1016/j.jconrel.2005.02.026

[24] M. Yang, S. K. Lai, Y. Y. Wang, W. X. Zhong, C. Happe, M. Zhang, J. Fu, J. Hanes, Biodegradable nanoparticles composed entirely of safe materials that rapidly penetrate human mucus. Angew. Chem. Int. Ed. 50, 2597–2600 (2011). 10.1002/anie.201006849 PMC3100893 21370345

[25] J. Fu, F. Sun, W. Liu, Y. Liu, M. Gedam, Q. Hu, C. Fridley, H. A. Quigley, J. Hanes, I. Pitha, Subconjunctival delivery of dorzolamide-loaded poly(ether-anhydride) microparticles produces sustained lowering of intraocular pressure in rabbits. Mol. Pharm. 13, 2987–2995 (2016). 27336794 10.1021/acs.molpharmaceut.6b00343 PMC5088785

[26] K. Hosseini, D. Matsushima, J. Johnson, G. Widera, K. Nyam, L. Kim, Y. Xu, Y. Yao, M. Cormier, Pharmacokinetic study of dexamethasone disodium phosphate using intravitreal, subconjunctival, and intravenous delivery routes in rabbits. J. Ocul. Pharmacol. Ther. 24, 301–308 (2008). 18476800 10.1089/jop.2007.0117

[27] X. Huang, Y. Duan, L. Zhao, S. Liu, D. Qin, F. Zhang, D. Lin, Dexamethasone pharmacokinetics characteristics via sub-tenon microfluidic system in uveitis rabbits. J. Drug Deliv. Sci. Technol. 57, 101639 (2020).

[28] M. N. Samtani, W. J. Jusko, Stability of dexamethasone sodium phosphate in rat plasma. Int. J. Pharm. 301, 262–266 (2005). 16054309 10.1016/j.ijpharm.2005.06.003 PMC4181326

[29] M. Salvi, Step-down steroid-sparing therapy in active thyroid eye disease. Nat. Rev. Endocrinol. 14, 634–635 (2018). 30262854 10.1038/s41574-018-0099-9

[30] D. Gupta, C. Illingworth, Treatments for corneal neovascularization: A review. Cornea 30, 927–938 (2011). 21389854 10.1097/ICO.0b013e318201405a

[31] A. K. Malkawi, K. H. Alzoubi, M. Jacob, G. Matic, A. Ali, A. Al Faraj, F. Almuhanna, M. Dasouki, A. M. Abdel Rahman, Metabolomics based profiling of dexamethasone side effects in rats. Front. Pharmacol. 9, 46 (2018). 29503615 10.3389/fphar.2018.00046 PMC5820529

[32] B. Kuley, P. P. Storey, M. Pancholy, A. Obeid, J. Murphy, J. Goodman, T. D. Wibbelsman, C. Regillo, A. Chiang, Ocular hypertension following intravitreal injection of 0.7mg dexamethasone implant versus 2mg triamcinolone. Semin. Ophthalmol. 35, 141–146 (2020). 32343619 10.1080/08820538.2020.1758161

[33] T. Yorio, G. C. Patel, A. F. Clark, Glucocorticoid-induced ocular hypertension: Origins and new approaches to minimize. Expert. Rev. Ophthalmol. 15, 145–157 (2020). 10.1080/17469899.2020.1762488 PMC10810227 38274668

[34] M. Oray, K. Abu Samra, N. Ebrahimiadib, H. Meese, C. S. Foster, Long-term side effects of glucocorticoids. Expert Opin. Drug Saf. 15, 457–465 (2016). 26789102 10.1517/14740338.2016.1140743

[35] D. Hos, M. Matthaei, F. Bock, K. Maruyama, M. Notara, T. Clahsen, Y. Hou, V. N. H. Le, A.-C. Salabarria, J. Horstmann, B. O. Bachmann, C. Cursiefen, Immune reactions after modern lamellar (DALK, DSAEK, DMEK) versus conventional penetrating corneal transplantation. Prog. Retin. Eye Res. 73, 100768 (2019). 31279005 10.1016/j.preteyeres.2019.07.001

[36] A. I. Vallelado, M. I. Lopez, M. Calonge, A. Sanchez, M. J. Alonso, Efficacy and safety of microspheres of cyclosporin A, a new systemic formulation, to prevent corneal graft rejection in rats. Curr. Eye Res. 24, 39–45 (2002). 12187493 10.1076/ceyr.24.1.39.5427

[37] V. Naageshwaran, V. P. Ranta, E. Toropainen, M. Tuomainen, G. Gum, E. Xie, S. Bhoopathy, A. Urtti, E. M. Del Amo, Topical pharmacokinetics of dexamethasone suspensions in the rabbit eye: Bioavailability comparison. Int. J. Pharm. 615, 121515 (2022). 35091006 10.1016/j.ijpharm.2022.121515

[38] G. F. Schwartz, H. A. Quigley, Adherence and persistence with glaucoma therapy. Surv. Ophthalmol. 53(Suppl. 1), S57–S68 (2008). 19038625 10.1016/j.survophthal.2008.08.002

[39] M. O. Price, A. Scanameo, M. T. Feng, F. W. Price, Descemet's membrane endothelial keratoplasty: Risk of immunologic rejection episodes after discontinuing topical corticosteroids. Ophthalmology 123, 1232–1236 (2016). 26983976 10.1016/j.ophtha.2016.02.001

[40] C. S. Jordan, M. O. Price, R. Trespalacios, F. W. Price, Graft rejection episodes after Descemet stripping with endothelial keratoplasty: Part one: Clinical signs and symptoms. Br. J. Ophthalmol. 93, 387–390 (2009). 19019931 10.1136/bjo.2008.140020

[41] X. W. Ng, K. L. Liu, A. B. Veluchamy, N. C. Lwin, T. T. Wong, S. S. Venkatraman, A biodegradable ocular implant for long-term suppression of intraocular pressure. Drug Deliv. Transl. Res. 5, 469–479 (2015). 26100093 10.1007/s13346-015-0240-4 PMC4551556

[42] E. M. Del Amo, A. K. Rimpela, E. Heikkinen, O. K. Kari, E. Ramsay, T. Lajunen, M. Schmitt, L. Pelkonen, M. Bhattacharya, D. Richardson, A. Subrizi, T. Turunen, M. Reinisalo, J. Itkonen, E. Toropainen, M. Casteleijn, H. Kidron, M. Antopolsky, K. S. Vellonen, M. Ruponen, A. Urtti, Pharmacokinetic aspects of retinal drug delivery. Prog. Retin. Eye Res. 57, 134–185 (2017). 28028001 10.1016/j.preteyeres.2016.12.001

[43] A. Y. Zhu, M. C. Marquezan, C. L. Kraus, C. R. Prescott, Pediatric corneal transplants: Review of current practice patterns. Cornea 37, 973–980 (2018). 29746327 10.1097/ICO.0000000000001613

[44] M. Banitt, R. K. Lee, Management of patients with combined glaucoma and corneal transplant surgery. Eye 23, 1972–1979 (2009). 19151651 10.1038/eye.2008.377

[45] S. Feizi, A. A. Azari, S. Safapour, Therapeutic approaches for corneal neovascularization. Eye Vis. 4,28(2017). 10.1186/s40662-017-0094-6 PMC5723406 29234686

[46] J. Yang, L. X. Luo, Y. M. Oh, T. Meng, G. H. Chai, S. Y. Xia, D. Emmert, B. Wang, C. G. Eberhart, S. Lee, W. J. Stark, L. M. Ensign, J. Hanes, Q. G. Xu, Sunitinib malate-loaded biodegradable microspheres for the prevention of corneal neovascularization in rats. J. Control. Release 327, 456–466 (2020). 32822742 10.1016/j.jconrel.2020.08.019 PMC8105765

[47] T. H. Dohlman, M. Omoto, J. Hua, W. Stevenson, S. M. Lee, S. K. Chauhan, R. Dana, VEGF-trap aflibercept significantly improves long-term graft survival in high-risk corneal transplantation. Transplantation 99, 678–686 (2015). 25606789 10.1097/TP.0000000000000512

[48] B. G. Short, Safety evaluation of ocular drug delivery formulations: Techniques and practical considerations. Toxicol. Pathol. 36, 49–62 (2008). 18337221 10.1177/0192623307310955

[49] K. Yamashita, S. Hatou, E. Inagaki, K. Higa, K. Tsubota, S. Shimmura, A rabbit corneal endothelial dysfunction model using endothelial-mesenchymal transformed cells. Sci. Rep. 8, 16868 (2018). 30442918 10.1038/s41598-018-35110-2 PMC6237874

[50] B. M. Gebhardt, W. Shi, Experimental corneal allograft rejection. Immunol. Res. 25, 1–26 (2002). 11868932 10.1385/IR:25:1:01

[51] M. Vézina, Comparative ocular anatomy in commonly used laboratory animals, in *Assessing Ocular Toxicology in Laboratory Animals*, A. B. Weir, M. Collins, Eds. (Humana Press, 2013), pp. 1–21.

[52] H. T. Hsueh, Y. C. Kim, I. Pitha, M. D. Shin, C. A. Berlinicke, R. T. Chou, E. Kimball, J. Schaub, S. Quillen, K. T. Leo, H. Han, A. Xiao, Y. Kim, M. Appell, U. Rai, H. Kwon, P. Kolodziejski, L. Ogunnaike, N. M. Anders, A. Hemingway, J. L. Jefferys, A. A. Date, C. Eberhart, T. V. Johnson, H. A. Quigley, D. J. Zack, J. Hanes, L. M. Ensign, Ion-complex microcrystal formulation provides sustained delivery of a multimodal kinase inhibitor from the subconjunctival space for protection of retinal ganglion cells. Pharmaceutics 13, 647 (2021). 34062883 10.3390/pharmaceutics13050647 PMC8147274

[53] OZURDEX [package insert] (Allergan Inc, 2009).

[54] NEOSPORIN [package insert] (Monarch Pharmaceuticals Inc, 2004).

[55] A. Beig, L. Feng, J. Walker, R. Ackermann, J. K. Y. Hong, T. Li, Y. Wang, B. Qin, S. P. Schwendeman, Physical-chemical characterization of octreotide encapsulated in commercial glucose-star PLGA microspheres. Mol. Pharm. 17, 4141–4151 (2020). 32876463 10.1021/acs.molpharmaceut.0c00619

[56] J. K. Y. Hong, S. P. Schwendeman, Characterization of octreotide–PLGA binding by isothermal titration calorimetry. Biomacromolecules 21, 4087–4093 (2020). 32885949 10.1021/acs.biomac.0c00885

[57] C. W. Wu, D. Ellenberg, J. H. Chang, Corneal angiogenesis and lymphangiogenesis, in *Ocular Disease* (W.B. Saunders, 2010), chap. 10, pp. 74–82.

