# Supplementary material for: Six-month effective treatment of corneal graft rejection
Source: Sci Adv. 2023 Mar 22;9(12):eadf4608. doi: 10.1126/sciadv.adf4608 (PMC10032610; doi:10.1126/sciadv.adf4608)
Supplement: Supplementary file 1 — Supplementary Materials and Methods Figs. S1 to S6 Tables S1 to S3 References [file sciadv.adf4608_sm.pdf]

Science Advances
AAAS

Supplementary Materials for

Six-month effective treatment of corneal graft rejection

Tuo Meng *et al.*

Corresponding author: Justin Hanes, hanes@jhmi.edu; Qingguo Xu, qxu@vcu.edu

Sci. Adv. **9**, eadf4608 (2023)
DOI: 10.1126/sciadv.adf4608

This PDF file includes:

Supplementary Materials and Methods
Figs. S1 to S6
Tables S1 to S3
References

Supplementary Materials and Methods

Polymer synthesis: Di-carboxyl-terminated PLA (PLA-2COOH) 5.1 kDa and 8.2 kDa were custom synthesized by Polymer Source, INC (Quebec, Canada). The scheme of polymer synthesis was showed as below:

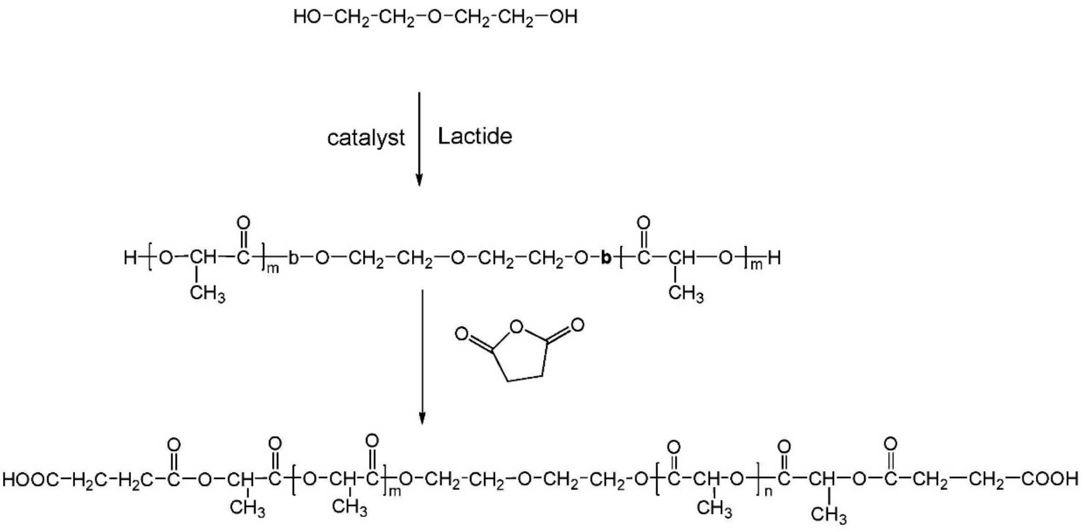

Scheme S1. The scheme of polymer synthesis

NMR: PLA-2COOH polymers were characterized by proton NMR (^{1}H NMR). Polymers were dissolved in deuterated chloroform. The ^{1}H NMR spectrum were obtained on Agilent DD2 400-MHz NMR Spectrometer (Santa Clara, CA) with MNova 10.0.1 (Mestrelab Research, Spain) processing software. The chemical shifts were represented in parts per million, with reference to the signal for $CDCl_3$ at 7.19 ppm.

GPC: The Mw and Mn of the PLA-2COOH polymers were analyzed by GPC using an Agilent 1260 Infinity II Triple Detector GPC system equipped with an isocratic pump with degasser (Agilent Technologies, Santa Clara, CA, USA). The Triple Detector System was equipped with multiangle laser light scattering detector, differential refractive index detector and a viscometer. Separations were performed using serially PLgel size exclusion columns (5µm-103 Å; 10µm-105 Å; 10µm-106 Å PLgel columns, 300 × 4.6 mm, Agilent Technologies, Santa Clara, CA, USA) at 35 °C using THF with triethylamine additive as the mobile phase at the flow rate of 1 ml/min. Polystyrene standards were applied for preparing calibration curves.

Determination of carboxyl content in the polymers: The content of terminal carboxyl groups in the PLA-2COOH polymers was quantified by potentiometric titration (*55, 56*). Briefly, the polymer was dissolved in acetonitrile/water (80/20, v/v) solution at 2.5 mg/ml. The polymer solution was titrated with 0.1 M sodium hydroxide solution dropwise until the pH of the system reached the plateau. The pH of the solution was monitored using a benchtop Fisher Scientific pH meter (MA, USA). The carboxyl content is expressed as µmol acid per gram polymer. Three replicates were conducted

for each polymer, and the results were expressed as mean ± SEM. The volume of base added was less than 9% of the total volume of the polymer solution to avoid changing the polymer pKa and precipitation of the polymer.

Measurement of DEX and DSP in rat plasma and ocular tissues: Ocular tissue samples were homogenized in 200 µl PBS before extraction using Next Advance Bullet Blender (Laboratory Supply Network, NH, USA). 50 µl of plasma or tissue homogenates were extracted with 150 µl of cold acetonitrile containing 5 ng/ml of the internal standard, dexamethasone-d5 (Cat No: D298802 Toronto Research Chemicals, Toronto, Canada). After centrifugation, the supernatant was collected for LC-MS/MS analysis. All ocular tissue samples were analyzed using a 1x PBS standard curve and plasma samples were analyzed using a plasma standard curve. DEX and DSP separation was achieved with an Agilent Zorbax XDB, C18 (2.1 × 50 mm, 3.5 µm) column at room temperature using a gradient with a flow rate of 0.3 mL/min. Mobile phase A was water containing 0.1% formic acid and mobile phase B was acetonitrile containing 0.1% formic acid. The gradient started with mobile phase B held at 10% for 1.0 minutes and increased to 100% over 1 minute; 100% mobile phase B was held for 1 minute and then returned back to 10% mobile phase B and allowed to equilibrate for 1 minute. The column effluent was monitored using a Sciex triple quadrupole™ 5500 mass-spectrometric detector (Sciex, Foster City, CA, USA) using electrospray ionization operating in positive mode. The spectrometer was programmed to monitor the following MRM transition 393.3 → 355.4 for dexamethasone (DEX), 473.0 → 435.0 for DSP and 393.3 → 360.1 for the internal standard, DEX-d5 (Toronto Research Chemicals, Toronto, Canada). The limit of quantification for DEX and DSP are 1ng/ml and 5 ng/ml respectively. Pharmacokinetic parameters were calculated from mean concentration-time data using non-compartmental methods in Phoenix WinNonlin version 8.3 (Certara, Princeton, NJ). The $AUC_{0\text{-}180\ days}$ was calculated using the log-linear trapezoidal method.

Electroretinogram (ERG): Rats were dark-adapted overnight, anesthetized by intramuscular injection of ketamine/xylazine, and placed on a heating pad set to 37 °C. The pupils were dilated with 1% tropicamide (Akron Pharmaceuticals, IL, USA) followed by 2.5% phenylephrine hydrochloride (Paragon BioTeck, OR, USA). A drop of 0.5% tetracaine hydrochloride eyedrop (Oceanside Pharmaceuticals, NJ, USA) was administered. After that, the 2.5% Hypromellose ophthalmic demulcent solution (Akron Pharmaceuticals, IL, USA) was applied followed by placing electrodes over the corneas. ERG responses were recorded by Espion ERG Diagnosys (MA, USA). Three intensities of lights (0.01, 0.1 and 1 cd.s/m^2) were tested for scotopic ERG first. Then two intensities of lights (3 and 10 cd.s/m^2) were measured for photopic ERG after a 10 min light adaptation. The a-wave and b-wave amplitudes were extracted using the Diagnosis Epsion software and transferred to Prism for analysis.

Results:

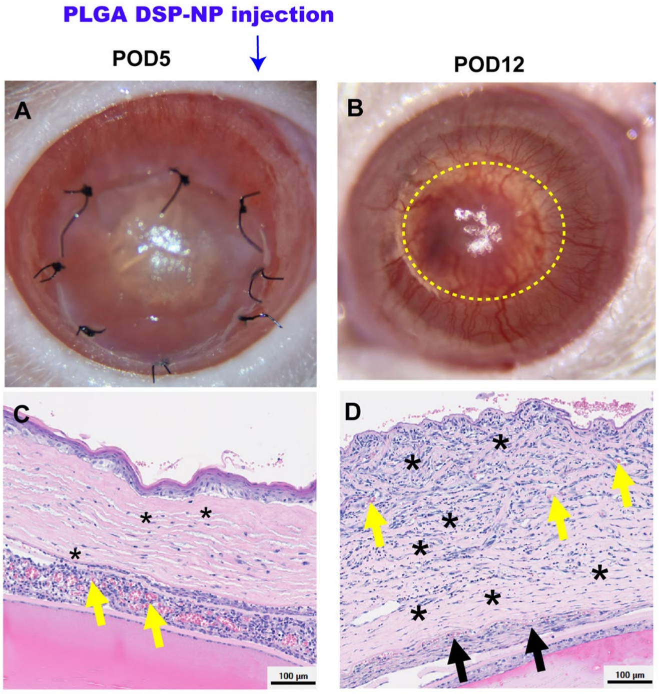

Fig. S1: PLGA DSP-NP treatment starting at POD5 was too late to rescue corneal graft rejection. (A) At POD5, corneal graft developed severe edema, opacity and neovascularization, and SCT injection of PLGA DSP-NP was administered to rescue corneal graft rejection. (B) At POD 12, cornea grafts presented aggravated opacity (score 3), edema (score 3) and intensive neovascularization (score 4) invading from the corneal bed to the graft with a total clinical score of 10 indicating the graft failure. H&E image of grafts at (C) POD 5 demonstrated an irregular corneal epithelia structure with inflammatory cell infiltration in the stroma layer (black asterisk) plus angiogenesis detected in the iris (Yellow arrow). (D) At POD 12, H&E analysis demonstrated overwhelming inflammatory cell infiltration in the corneal stroma and neovascularization was sprouting from limbus to the stroma layer of the corneal graft (Yellow arrow). Inflammatory pannus was also shown (Black arrow), further indicating the infiltration of lymphocytes and neovascularization in the corneal grafts (*57*).

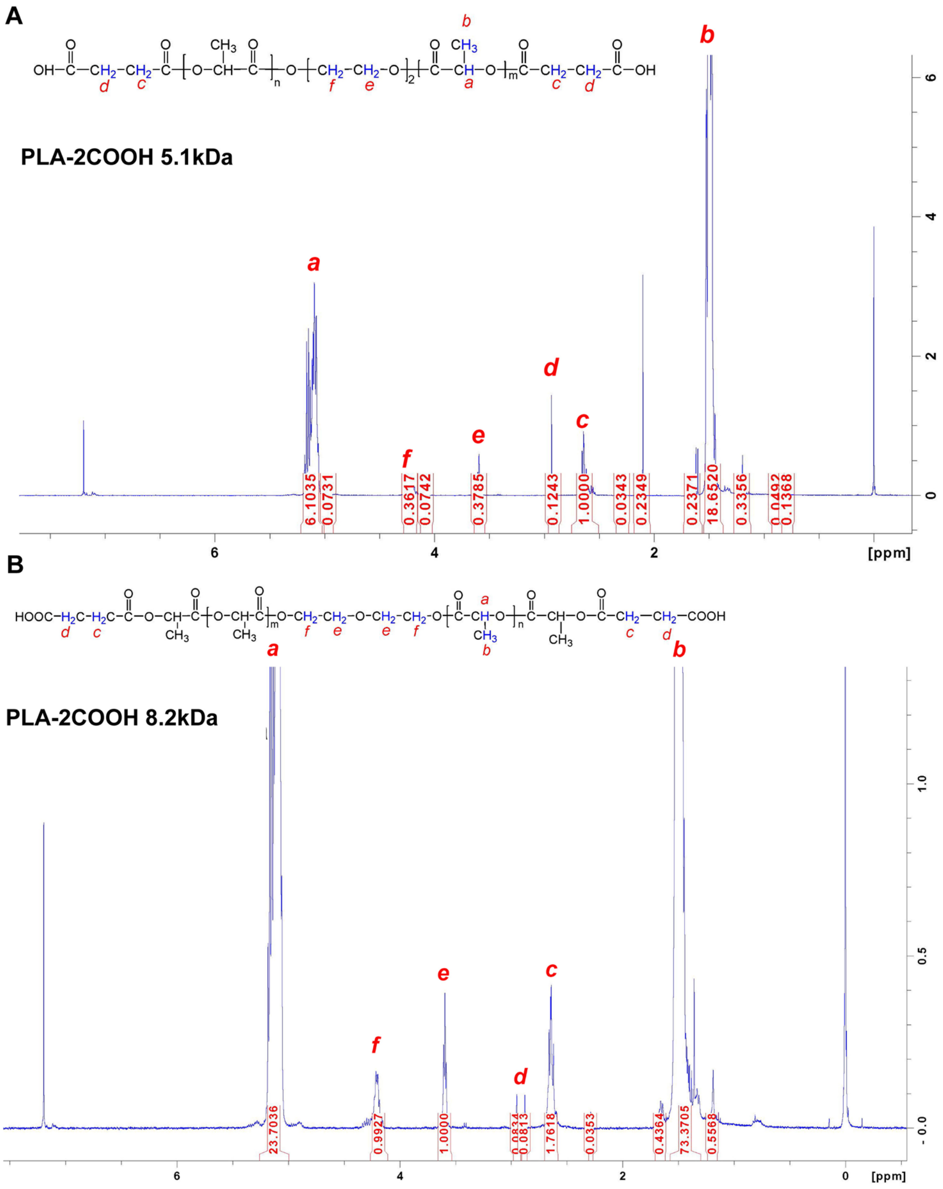

Fig. S2: ^{1}H NMR spectrum of (A) PLA-2COOH 5.1 kDa and (B) PLA-2COOH 8.2 kDa. The peaks at 2.7 ppm correspond to the hydrogen atoms of the methylene group close to the carboxylic acid ends in both PLA polymers. The peaks at 5.1 ppm and 1.5 ppm indicate the hydrogen atoms of the methine and methyl groups on the replicate parts of the PLA polymers, respectively. In addition, the peak of 3.6 ppm and 4.2 ppm correspond to the protons of methylene groups of ethylene glycol units.

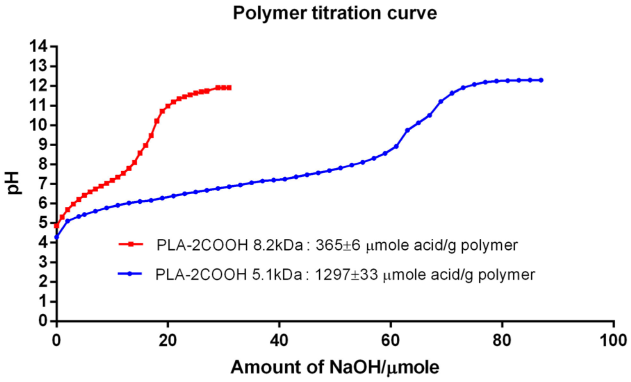

Fig. S3: Representative titration curve of PLA-2COOH polymers.

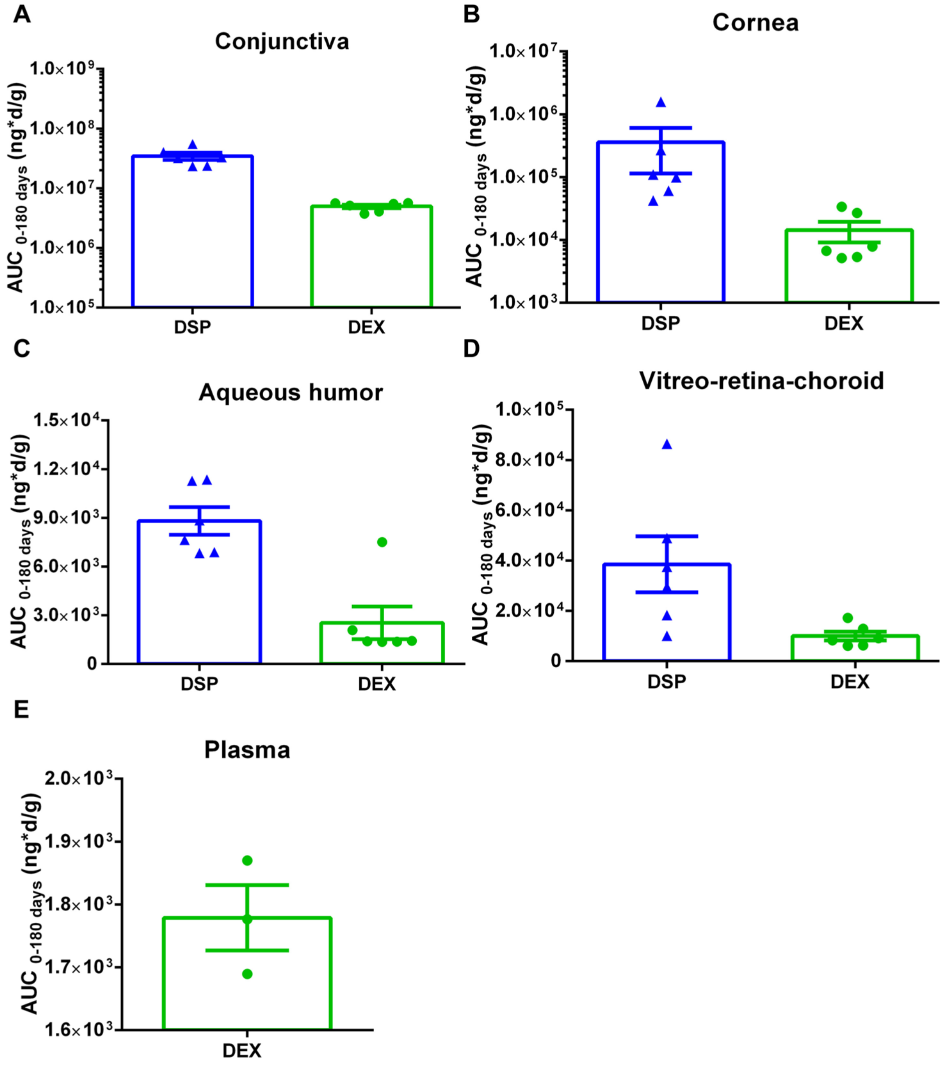

Fig. S4: AUC for PK study. AUC for DSP and/or DEX over 6 months ($AUC_{0\text{-}180\ days}$) after a single SCT injection of 600 µg PLA DSP-NP. (A) conjunctiva, (B) cornea, (C) aqueous humor, (D) vitreo-retina-choroid, and (E) plasma. AUC calculations were performed under non-compartment analysis using WinNonLin software. Data are presented as mean ± SEM (n = 6 for ocular tissues, n = 3 for plasma samples).

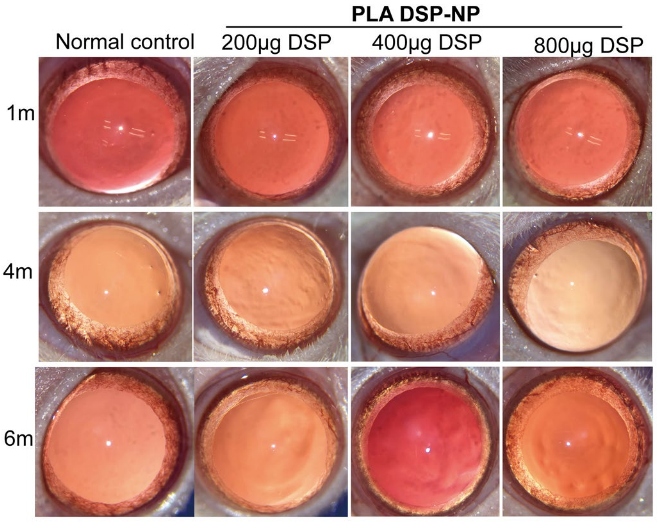

Fig. S5: Ocular images at 1 m, 4 m and 6 m after PLA DSP-NP administration. Healthy SD rats received a single SCT injection of PLA DSP-NP at doses of 200 μg DSP, 400 μg DSP and 800 μg DSP. At 1 m, 4 m and 6 m after administration, ocular images were taken, and no cataract was detected.

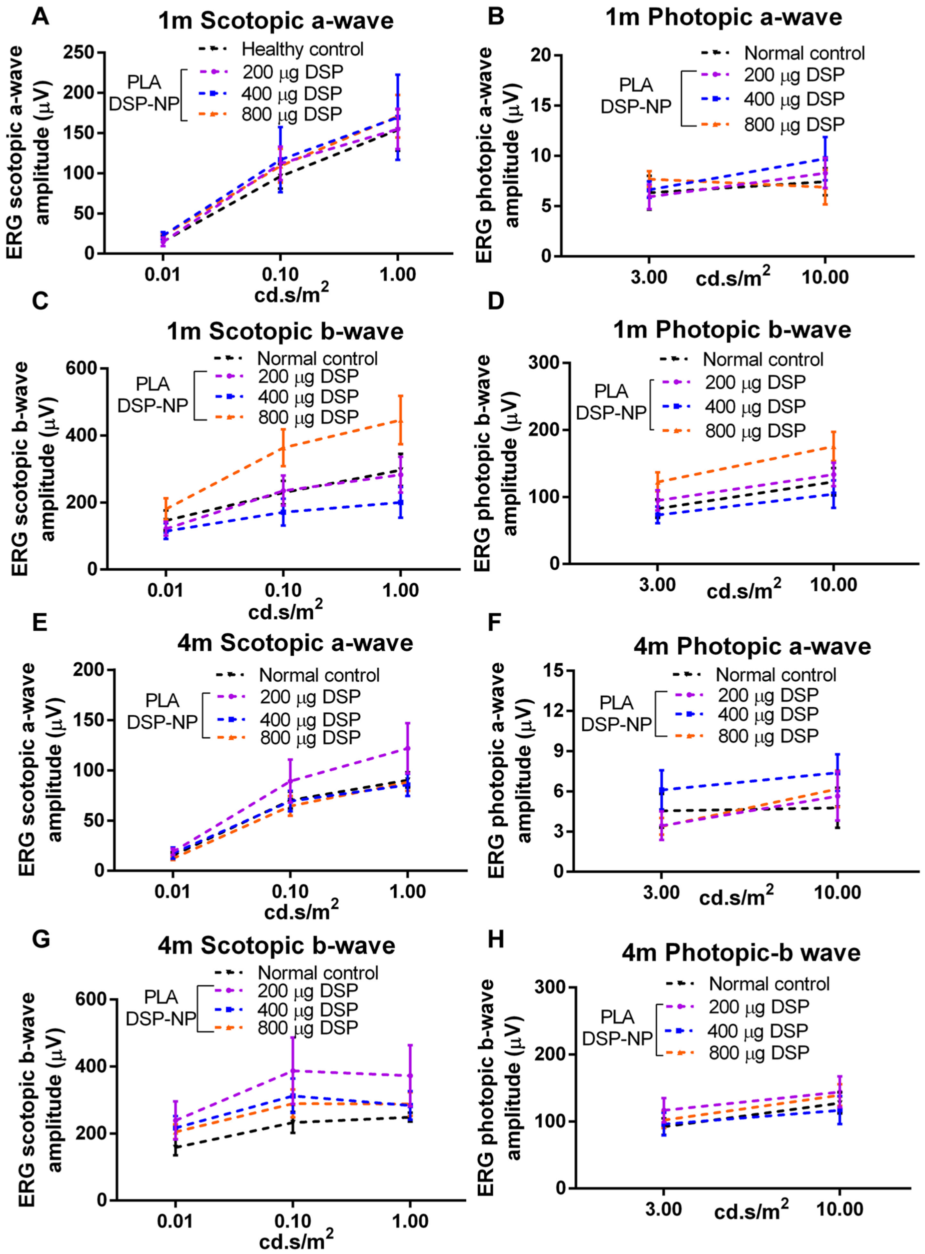

Fig. S6: ERG at 1 month and 4 months after PLA DSP-NP administration. Quantification of amplitudes of (A, E) scotopic a-wave, (B, F) photopic a-wave, (C, G) scotopic b-wave, (B, F) photopic a-wave and (D, H) photopic b-wave at 1 m and 4 m after PLA DSP-NP injection. Statistical analysis for (A~H): Two-way ANOVA followed by Dunnett post hoc test. (* for compare with healthy control: $^*p \leq 0.05$, ** $p \leq 0.01$, *** $p \leq 0.001$. n=5-6 for ERG studies).

Table S1: GPC characterization of PLA-2COOH polymers.

Polymer	Number average molecular weight (M_n) g/mol	Weight average molecular weight (M_w) g/mol	PDI
PLA-2COOH 5.1 kDa	3741	5105	1.36
PLA-2COOH 8.2 kDa	6500	8200	1.25

Table S2: Primer sequences used in RT-PCR analysis.

Gene	Forward primer (5'~3')	Reverse primer (5'~3')
TNF-α	GACCCTCACACTCAGATCATCTTCT	TGCTACGACGTGGGCTACG
IL-1β	CCCTGCAGCTGGAGAGTGTGG	TGTGCTCTGCTTGAGAGGTGCT
IL-4	ACAGGAGAAGGGACGCCAT	GAAGCCCTACAGACGAGCTCA
INF-γ	CACGCCGCGTCTTGGT	TCTAGGCTTTCAATGAGTGTGCC
Granzyme B	TGTGCTATGTGGCTGGTTGG	TTTGATCTTTGGGTCCCCTG

Table S3: Clinical parameters evaluation (score: 0~4) (*17*).

Score	Transparency	Neovascularization	Edema
0	Clear	None	None
1	Slightly opacity	Neovascularization invading to 1/3 of recipient	Slight
2	Mild opacity with iris details visible	Neovascularization invading to 2/3 of recipient	Moderate stromal edema
3	Moderate opacity, iris details not visible	Neovascularization invading to the edge of graft	Marked stromal edema
4	Serve opacity, white cornea	Neovascularization invading to the graft	Serve edema

REFERENCES

1. World Health Organization, Blindness and vision impairment (2020); www.who.int/news-room/fact-sheets/detail/blindness-and-visual-impairment.

2. D. T. H. Tan, J. K. G. Dart, E. J. Holland, S. Kinoshita, Corneal transplantation. *Lancet* **379**, 1749–1761 (2012).

3. T. M. M. Ways, W. M. Lau, V. V. Khutoryanskiy, Chitosan and its derivatives for application in mucoadhesive drug delivery systems. *Polymers* **10**, 267 (2018).

4. P. Gain, R. Jullienne, Z. He, M. Aldossary, S. Acquart, F. Cognasse, G. Thuret, Global Survey of Corneal Transplantation and Eye Banking. *JAMA Ophthalmol.* **134**, 167–173 (2016).

5. J. Yin, Advances in corneal graft rejection. *Curr. Opin. Ophthalmol.* **32**, 331–337 (2021).

6. Y. Qazi, P. Hamrah, Corneal allograft rejection: Immunopathogenesis to therapeutics. *J. Clin. Cell Immunol.* **2013** (Suppl. 9), 006 (2013).

7. T. B. Abud, A. Di Zazzo, A. Kheirkhah, R. Dana, Systemic immunomodulatory strategies in high-risk corneal transplantation. *J. Ophthalmic Vis. Res.* **12**, 81–92 (2017).

8. I. Ezon, C. Y. Shih, L. M. Rosen, T. Suthar, I. J. Udell, Immunologic graft rejection in descemet's stripping endothelial keratoplasty and penetrating keratoplasty for endothelial disease. *Ophthalmology* **120**, 1360–1365 (2013).

9. S. A. Gaballa, U. B. Kompella, O. Elgarhy, A. M. Alqahtani, B. Pierscionek, R. G. Alany, H. Abdelkader, Corticosteroids in ophthalmology: Drug delivery innovations, pharmacology, clinical applications, and future perspectives. *Drug. Deliv. Transl. Res.* **11**, 866–893 (2020).

10. F. W. Price Jr.., D. A. Price, V. Ngakeng, M. O. Price, Survey of steroid usage patterns during and after low-risk penetrating keratoplasty. *Cornea* **28**, 865–870 (2009).

11. E. Guilbert, J. Bullet, O. Sandali, E. Basli, L. Laroche, V. M. Borderie, Long-term rejection incidence and reversibility after penetrating and lamellar keratoplasty. *Am. J. Ophthalmol.*

155, 560–569.e2 (2013).

12. W. J. Stark, M. G. Maguire; The Collaborative Corneal Transplantation Studies Research Group, Design and methods of the collaborative corneal transplantation studies. *Cornea* **12**, 93–103 (1993).

13. M. R. Razeghinejad, L. J. Katz, Steroid-induced iatrogenic glaucoma. *Ophthalmic Res.* **47**, 66–80 (2011).

14. G. Roberti, F. Oddone, L. Agnifili, A. Katsanos, M. Michelessi, L. Mastropasqua, L. Quaranta, I. Riva, L. Tanga, G. Manni, Steroid-induced glaucoma: Epidemiology, pathophysiology, and clinical management. *Surv. Ophthalmol.* **65**, 458–472 (2020).

15. O. Weijtens, E. J. Feron, R. C. Schoemaker, A. F. Cohen, E. G. W. M. Lentjes, F. P. H. T. M. Romijn, J. C. van Meurs, High concentration of dexamethasone in aqueous and vitreous after subconjunctival injection. *Am. J. Ophthalmol.* **128**, 192–197 (1999).

16. J. B. Randleman, R. D. Stulting, Prevention and treatment of corneal graft rejection: Current practice patterns (2004). *Cornea* **25**, 286–290 (2006).

17. Q. Pan, Q. Xu, N. J. Boylan, N. W. Lamb, D. G. Emmert, J. C. Yang, L. Tang, T. Heflin, S. Alwadani, C. G. Eberhart, W. J. Stark, J. Hanes, Corticosteroid-loaded biodegradable nanoparticles for prevention of corneal allograft rejection in rats. *J. Control. Release* **201**, 32–40 (2015).

18. L. Luo, J. Yang, Y. Oh, M. J. Hartsock, S. Xia, Y.-C. Kim, Z. Ding, T. Meng, C. G. Eberhart, L. M. Ensign, J. E. Thorne, W. J. Stark, E. J. Duh, Q. Xu, J. Hanes, Controlled release of corticosteroid with biodegradable nanoparticles for treating experimental autoimmune uveitis. *J. Control. Release* **296**, 68–80 (2019).

19. V. S. Gorantla, J. H. Barker, J. W. Jones, Jr., K. Prabhune, C. Maldonado, D. K. Granger, Immunosuppressive agents in transplantation: Mechanisms of action and current anti-rejection strategies. *Microsurgery* **20**, 420–429 (2000).

20. R. I. Lechler, M. Sykes, A. W. Thomson, L. A. Turka, Organ transplantation—How much of the promise has been realized? *Nat. Med.* **11**, 605–613 (2005).

21. M. R. Prausnitz, J. S. Noonan, Permeability of cornea, sclera, and conjunctiva: A literature analysis for drug delivery to the eye. *J. Pharm. Sci.* **87**, 1479–1488 (1998).

22. T. Ishihara, M. Takahashi, M. Higaki, Y. Mizushima, Efficient encapsulation of a water-soluble corticosteroid in biodegradable nanoparticles. *Int. J. Pharm.* **365**, 200–205 (2009).

23. T. Ishihara, N. Izumo, M. Higaki, E. Shimada, T. Hagi, L. Mine, Y. Ogawa, Y. Mizushima, Role of zinc in formulation of PLGA/PLA nanoparticles encapsulating betamethasone phosphate and its release profile. *J. Control. Release* **105**, 68–76 (2005).

24. M. Yang, S. K. Lai, Y. Y. Wang, W. X. Zhong, C. Happe, M. Zhang, J. Fu, J. Hanes, Biodegradable nanoparticles composed entirely of safe materials that rapidly penetrate human mucus. *Angew. Chem. Int. Ed.* **50**, 2597–2600 (2011).

25. J. Fu, F. Sun, W. Liu, Y. Liu, M. Gedam, Q. Hu, C. Fridley, H. A. Quigley, J. Hanes, I. Pitha, Subconjunctival delivery of dorzolamide-loaded poly(ether-anhydride) microparticles produces sustained lowering of intraocular pressure in rabbits. *Mol. Pharm.* **13**, 2987–2995 (2016).

26. K. Hosseini, D. Matsushima, J. Johnson, G. Widera, K. Nyam, L. Kim, Y. Xu, Y. Yao, M. Cormier, Pharmacokinetic study of dexamethasone disodium phosphate using intravitreal, subconjunctival, and intravenous delivery routes in rabbits. *J. Ocul. Pharmacol. Ther.* **24**, 301–308 (2008).

27. X. Huang, Y. Duan, L. Zhao, S. Liu, D. Qin, F. Zhang, D. Lin, Dexamethasone pharmacokinetics characteristics via sub-tenon microfluidic system in uveitis rabbits. *J. Drug Deliv. Sci. Technol.* **57**, 101639 (2020).

28. M. N. Samtani, W. J. Jusko, Stability of dexamethasone sodium phosphate in rat plasma. *Int. J. Pharm.* **301**, 262–266 (2005).

29. M. Salvi, Step-down steroid-sparing therapy in active thyroid eye disease. *Nat. Rev. Endocrinol.* **14**, 634–635 (2018).

30. D. Gupta, C. Illingworth, Treatments for corneal neovascularization: A review. *Cornea* **30**, 927–938 (2011).

31. A. K. Malkawi, K. H. Alzoubi, M. Jacob, G. Matic, A. Ali, A. Al Faraj, F. Almuhanna, M. Dasouki, A. M. Abdel Rahman, Metabolomics based profiling of dexamethasone side effects in rats. *Front. Pharmacol.* **9**, 46 (2018).

32. B. Kuley, P. P. Storey, M. Pancholy, A. Obeid, J. Murphy, J. Goodman, T. D. Wibbelsman, C. Regillo, A. Chiang, Ocular hypertension following intravitreal injection of 0.7mg dexamethasone implant versus 2mg triamcinolone. *Semin. Ophthalmol.* **35**, 141–146 (2020).

33. T. Yorio, G. C. Patel, A. F. Clark, Glucocorticoid-induced ocular hypertension: Origins and new approaches to minimize. *Expert. Rev. Ophthalmol.* **15**, 145–157 (2020).

34. M. Oray, K. Abu Samra, N. Ebrahimiadib, H. Meese, C. S. Foster, Long-term side effects of glucocorticoids. *Expert Opin. Drug Saf.* **15**, 457–465 (2016).

35. D. Hos, M. Matthaei, F. Bock, K. Maruyama, M. Notara, T. Clahsen, Y. Hou, V. N. H. Le, A.-C. Salabarria, J. Horstmann, B. O. Bachmann, C. Cursiefen, Immune reactions after modern lamellar (DALK, DSAEK, DMEK) versus conventional penetrating corneal transplantation. *Prog. Retin. Eye Res.* **73**, 100768 (2019).

36. A. I. Vallelado, M. I. Lopez, M. Calonge, A. Sanchez, M. J. Alonso, Efficacy and safety of microspheres of cyclosporin A, a new systemic formulation, to prevent corneal graft rejection in rats. *Curr. Eye Res.* **24**, 39–45 (2002).

37. V. Naageshwaran, V. P. Ranta, E. Toropainen, M. Tuomainen, G. Gum, E. Xie, S. Bhoopathy, A. Urtti, E. M. Del Amo, Topical pharmacokinetics of dexamethasone suspensions in the rabbit eye: Bioavailability comparison. *Int. J. Pharm.* **615**, 121515 (2022).

38. G. F. Schwartz, H. A. Quigley, Adherence and persistence with glaucoma therapy. *Surv.*

Ophthalmol. **53** (Suppl. 1), S57–S68 (2008).

39. M. O. Price, A. Scanameo, M. T. Feng, F. W. Price, Descemet's membrane endothelial keratoplasty: Risk of immunologic rejection episodes after discontinuing topical corticosteroids. *Ophthalmology* **123**, 1232–1236 (2016).

40. C. S. Jordan, M. O. Price, R. Trespalacios, F. W. Price, Graft rejection episodes after Descemet stripping with endothelial keratoplasty: Part one: Clinical signs and symptoms. *Br. J. Ophthalmol.* **93**, 387–390 (2009).

41. X. W. Ng, K. L. Liu, A. B. Veluchamy, N. C. Lwin, T. T. Wong, S. S. Venkatraman, A biodegradable ocular implant for long-term suppression of intraocular pressure. *Drug Deliv. Transl. Res.* **5**, 469–479 (2015).

42. E. M. Del Amo, A. K. Rimpela, E. Heikkinen, O. K. Kari, E. Ramsay, T. Lajunen, M. Schmitt, L. Pelkonen, M. Bhattacharya, D. Richardson, A. Subrizi, T. Turunen, M. Reinisalo, J. Itkonen, E. Toropainen, M. Casteleijn, H. Kidron, M. Antopolsky, K. S. Vellonen, M. Ruponen, A. Urtti, Pharmacokinetic aspects of retinal drug delivery. *Prog. Retin. Eye Res.* **57**, 134–185 (2017).

43. A. Y. Zhu, M. C. Marquezan, C. L. Kraus, C. R. Prescott, Pediatric corneal transplants: Review of current practice patterns. *Cornea* **37**, 973–980 (2018).

44. M. Banitt, R. K. Lee, Management of patients with combined glaucoma and corneal transplant surgery. *Eye* **23**, 1972–1979 (2009).

45. S. Feizi, A. A. Azari, S. Safapour, Therapeutic approaches for corneal neovascularization. *Eye Vis.* **4**, 28 (2017).

46. J. Yang, L. X. Luo, Y. M. Oh, T. Meng, G. H. Chai, S. Y. Xia, D. Emmert, B. Wang, C. G. Eberhart, S. Lee, W. J. Stark, L. M. Ensign, J. Hanes, Q. G. Xu, Sunitinib malate-loaded biodegradable microspheres for the prevention of corneal neovascularization in rats. *J. Control. Release* **327**, 456–466 (2020).

47. T. H. Dohlman, M. Omoto, J. Hua, W. Stevenson, S. M. Lee, S. K. Chauhan, R. Dana, VEGF-trap aflibercept significantly improves long-term graft survival in high-risk corneal transplantation. *Transplantation* **99**, 678–686 (2015).

48. B. G. Short, Safety evaluation of ocular drug delivery formulations: Techniques and practical considerations. *Toxicol. Pathol.* **36**, 49–62 (2008).

49. K. Yamashita, S. Hatou, E. Inagaki, K. Higa, K. Tsubota, S. Shimmura, A rabbit corneal endothelial dysfunction model using endothelial-mesenchymal transformed cells. *Sci. Rep.* **8**, 16868 (2018).

50. B. M. Gebhardt, W. Shi, Experimental corneal allograft rejection. *Immunol. Res.* **25**, 1–26 (2002).

51. M. Vézina, Comparative ocular anatomy in commonly used laboratory animals, in *Assessing Ocular Toxicology in Laboratory Animals*, A. B. Weir, M. Collins, Eds. (Humana Press, 2013), pp. 1–21.

52. H. T. Hsueh, Y. C. Kim, I. Pitha, M. D. Shin, C. A. Berlinicke, R. T. Chou, E. Kimball, J. Schaub, S. Quillen, K. T. Leo, H. Han, A. Xiao, Y. Kim, M. Appell, U. Rai, H. Kwon, P. Kolodziejski, L. Ogunnaike, N. M. Anders, A. Hemingway, J. L. Jefferys, A. A. Date, C. Eberhart, T. V. Johnson, H. A. Quigley, D. J. Zack, J. Hanes, L. M. Ensign, Ion-complex microcrystal formulation provides sustained delivery of a multimodal kinase inhibitor from the subconjunctival space for protection of retinal ganglion cells. *Pharmaceutics* **13**, 647 (2021).

53. OZURDEX [package insert] (Allergan Inc, 2009).

54. NEOSPORIN [package insert] (Monarch Pharmaceuticals Inc, 2004).

55. A. Beig, L. Feng, J. Walker, R. Ackermann, J. K. Y. Hong, T. Li, Y. Wang, B. Qin, S. P. Schwendeman, Physical-chemical characterization of octreotide encapsulated in commercial glucose-star PLGA microspheres. *Mol. Pharm.* **17**, 4141–4151 (2020).

56. J. K. Y. Hong, S. P. Schwendeman, Characterization of octreotide–PLGA binding by isothermal titration calorimetry. *Biomacromolecules* **21**, 4087–4093 (2020).

57. C. W. Wu, D. Ellenberg, J. H. Chang, Corneal angiogenesis and lymphangiogenesis, in *Ocular Disease* (W.B. Saunders, 2010), chap. 10, pp. 74–82.
